# Acute and Sub-chronic Toxicity of Indole Alkaloids Extract from Leaves of *Alstonia scholaris* (L.) R. Br. in Beagle Dogs

**DOI:** 10.1007/s13659-020-00246-0

**Published:** 2020-06-10

**Authors:** Yun-Li Zhao, Min Su, Jian-Hua Shang, Xia Wang, Guang-Lei Bao, Jia Ma, Qing-Di Sun, Fang Yuan, Jing-Kun Wang, Xiao-Dong Luo

**Affiliations:** 1 State Key Laboratory of Phytochemistry and Plant Resources in West China, Kunming Institute of Botany, Chinese Academy of Sciences, Kunming, 650201 People’s Republic of China; 2 Yunnan Institute of Medical Material, Kunming, 650111 People’s Republic of China; 3 Key Laboratory of Medicinal Chemistry for Natural Resource, Ministry of Education and Yunnan Province, School of Chemical Science and Technology, Yunnan University, Kunming, 650091 People’s Republic of China; 4 Jiangsu Nhwa Pharmaceutical Co., Ltd, Xuzhou, 221009 People’s Republic of China

**Keywords:** *Alstonia scholaris*, Indole alkaloids, Acute toxicity, Sub-chronic toxicity, Beagle dog, Non-observed-adverse-effect-level

## Abstract

**Electronic supplementary material:**

The online version of this article (10.1007/s13659-020-00246-0) contains supplementary material, which is available to authorized users.

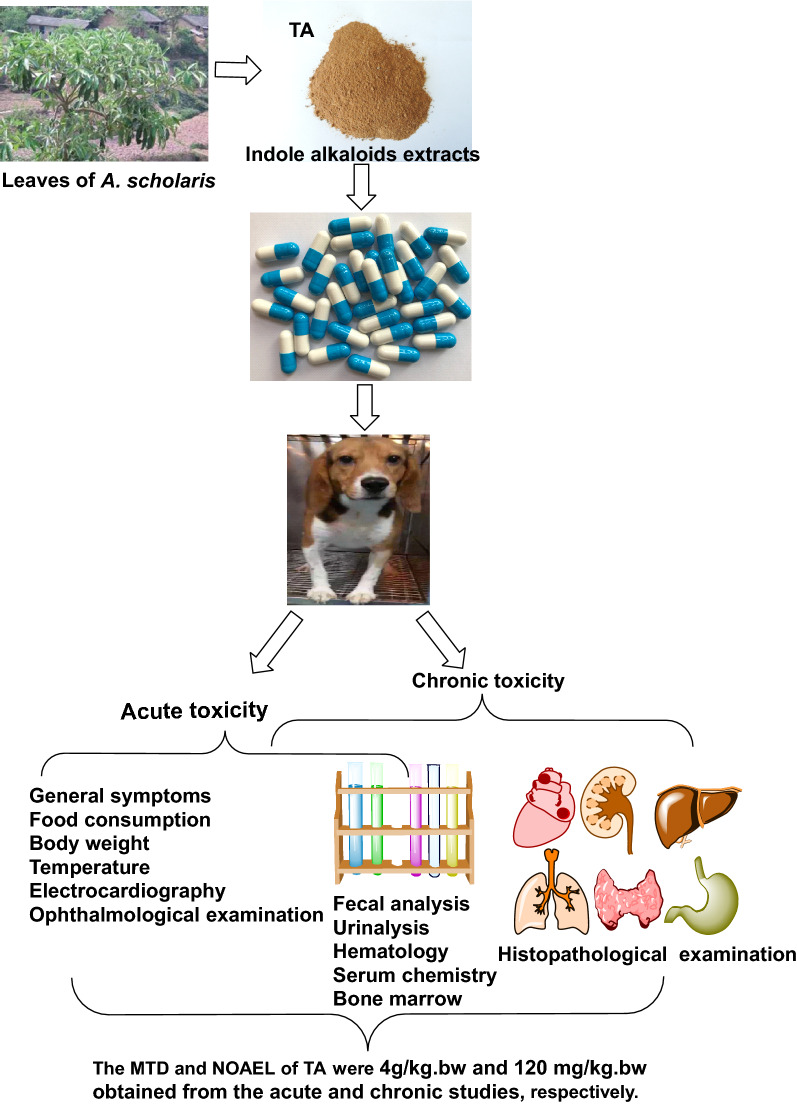

## Introduction

*Alstonia scholaris* (L.) R. Br. is widely distributed in tropical and subtropical mountains and evergreen or valley rain forests of Africa and Asia [1]. The leaves are traditionally used for management of respiratory ailments in Dai medicine practiced in Yunnan Province, China [2]. Over the years, our team has extensively investigated the phytochemical constituents of different parts of the plant [3–22]. Among the compounds isolated, eight monoterpenoid indole alkaloids from *A. scholaris* have been selected as “hot off the press” compounds, as specified in *Natural Products Report* (supporting information, Table S1). Moreover, Sixteen *Alstonia* alkaloids were total synthesized by chemists after their structures or bioactivities were published (supporting information, Table S2).

Beyond that, previous pharmacological studies have confirmed that the indole alkaloids extract from leaves of *A. scholaris* have antitussive, anti-asthmatic, expectorant [23], analgesic, anti-inflammatory [24], anti-airway inflammation [25], and anti-allergic effects in response to ovalbumin [26], provide protection against LPS-induced post-infectious cough [27] and porcine pancreatic elastase caused emphysema in vivo [28]. Alkaloids have been shown to trigger activation of β_2_ adrenergic receptors [29] and inhibit nuclear factor-κB bioactivity in vitro [30].

Given that TA contains a series of novel compounds with prominent pharmacological activity in respiratory disease correlated to systemic exposure, its pharmacokinetics profile has been extensively examined to establish the major active components in animals and humans. The pharmacokinetic properties of rats indicated T_*max*_ within 10 min for scholaricine and 19-epischolaricine and 15 min for vallesamine and picrinine, followed by elimination from plasma after 24 h. Moreover, results indicated that vallesamine and picrinine had a very rapid effect while scholaricine and 19-epischolaricine were faster onset time in vivo [26].

The metabolic pathways of four representative alkaloids in rats mainly involved hydroxylation and glucuronidation reactions. Furthermore, 33 compounds in plasma, 40 in urine, and 38 in feces were identified and scholaricine-type alkaloids shown to enter the circulation more readily than the other types [31]. In parallel with the above findings, in eligible healthy Chinese volunteers administered oral TA capsules at different doses (20, 40, 80 or 120 mg), AUC exposure was in the order: vallesamine > scholaricine > 19-epischolaricine > picrinine. Different patterns were observed whereby 19-epischolaricine, vallesamine and picrinine exhibited linear pharmacokinetic characteristics while scholaricine conformed to non-linear pharmacokinetics [32]. Based on the chemical profiles and metabolites of alkaloid extracts of leaves of *A. scholaris*, scholaricine, 19-epischolaricine, vallesamine and picrinine have been determined as the major indole alkaloids (Fig. S1A).

Despite the known chemical composition, substantial pharmacological effects and metabolic characteristics of TA, related toxicological studies such as acute toxicity test in mice and chronic in rats [33] as well as genotoxicity [34] have been studied by our group. Therefore, as part of a comprehensive preclinical program, toxicological assessment of TA and estimation of the appropriate doses in nonrodents are essential. In the current study, acute and sub-chronic oral toxicity of TA was investigated using beagle dogs. Clinical observations, the analysis of hematology and serum biochemistry, urinalysis, fecal examination, ophthalmoscopy, electrocardiography, bone marrow and histopathological analyses were performed at the Drug Safety Evaluation Center of Yunnan Institute of Medical Material (Yunnan Province, China) in compliance with the China Food and Drug Administration (CFDA) Good Laboratory Practice Regulations [35].

## Results

### Acute Oral Toxicity Test in Dogs

As shown in Table 1, after single oral administration of total alkaloids, both male and female dogs displayed treatment-related emesis beginning approximately 20 min post-dosage, but not after 40 min of administration. Additionally, reddening of perioral mucosa, limbs and ears was observed from approximately 30 min, but not evident at 3 h after administration. In particular, local swelling of ear initially occurred from ~ 20 min of treatment but was not evident after 3 h. We additionally observed unsteady walking and drooling in one male dog at ~ 40 min, but disappeared after 8 min post-administration.

**Table 1:** Evaluation of acute toxicity of indole alkaloids derived from *A. scholaris* orally administered in beagle dogs

Animals	Symptoms	Occurrence time	Disappeared time	Lasting time	Notes
No.: 62244 M Male Administration time: 9:00	Unsteady walking	9:42	9:50	8 min	–
	Drooling	10:00	10:05	5 min	–
	Emesis	9:40	10:25	45 min	The vomit was a yellowish capsule, and 6 episodes of vomiting occurred
	Reddening of perioral mucosa, limbs and ears	9:45	12:45	3 h	–
	Local swelling of right hind foot	9:40	12:41	3 h	–
No.: 71192F Female Administration time: 10:36	Emesis	10:54	11:24	30 min	The vomit was a yellowish capsule, and 7 episodes of vomiting occurred
	Reddening of perioral mucosa, limbs and ears	11:06	12:25	79 min	–
	Local swelling of ear	10:54	12:30	96 min	–

“–” Signifies that the symptoms were mild and temporal

No mortality was induced by TA in this study. Review of individual data on body weight, rectal temperature, hematology, biochemical indices, ECG, urine and fecal parameters disclosed normal physiological variations but no significant changes attributable to TA administration. Detailed individual in-life animal data are presented in supporting information (Tables S3-S10).

### General Observation of Sub-chronic Oral Toxicity in dogs

During the treatment period, one female (control group) and male (120 mg/kg.bw) dog displayed partial hair loss in the joint on week 11. Two dogs (one female and one male) of the 20 mg/kg.bw group had loose feces from weeks 4 to 12. Two male animals from the 60 mg/kg.bw treatment group displayed occasional loose feces at weeks 6 and 7. In one male and two female dogs of the 120 mg/kg.bw group, green viscous material in feces was observed at weeks 11 and 12. In addition, animals of TA groups displayed vomiting episodes one week after administration, accompanied by drooling in the 120 mg/kg.bw group, which disappeared at the end of the treatment period. During the recovery period, one female dog in the 120 mg/kg.bw group displayed swelling around the nipples, which was potentially related to the physiological cycle of the animal and not TA administration. All the above symptoms were incidental in individual animals and no dose–response relationship was evident, and may therefore be wholly unrelated to TA administration. No mortality was observed during this study.

### Food Intake, Body Weight, Rectal Temperature, and Ophthalmological Examination

As shown in supporting information, no significant differences in food intake (Table S11), body weights (Table S12), rectal temperatures (Table S13) and ophthalmological parameters (Tables S14–16) were recorded between the control and TA treatment groups during the study period. The rectal temperature of the 20 mg/kg.bw group on weeks 5 and 9 was slightly lower than that before administration (*p* < 0.05/0.01), determined through self-comparison, but fluctuated within the normal range (38.0–39.5 °C), indicating no real biological significance.

### Urinalysis Results

Urinalysis was evaluated via self-comparison and differences among the four groups assessed (Table S17–22). Individual animals of the control and TA-treated groups displayed positivity for urine glucose (GLU), uric bilirubin (BIL), ketone bodies (KET), urine occult blood (BLO), urine protein (PRO) and white blood cells (WBC), with no significant differences (*p* > 0.05). The pH values at weeks 5, 9 and 13 of the 60 mg/kg.bw treatment group and URO level following withdrawal of TA for 4 weeks of the 120 mg/kg.bw group were significantly increased (*p* < 0.05/0.01, Table S23) while urine SG at week 5 in the 20 and 60 mg/kg.bw groups showed a relatively small but significant decrease (*p* < 0.05). However, no obvious dose–response or time-response relationship was observed.

### Fecal Analysis

Fecal occult blood (OB) in control and TA-treated animals was occasionally positive before the treatment period. No significant differences in OB and parasitic ovum parameters were evident between the groups during the administration period and end of the recovery period (Table S24).

### Lead Electrocardiographic (ECG) and Bone Marrow Examinations

After 13 weeks of administration of TA to beagle dogs, no obvious TA-related abnormalities were observed on the electrocardiogram (assessing arrhythmia, specific S-T, T, and P wave) and myelogram. We observed no significant differences between the control and TA-treated groups (*p* > 0.05). Summary data for measured ECG parameters are presented in Table S25 and myelography data in Table S26.

### Hematological and Biochemical Parameters

No test article-related effects were noted in terms of hematological (Tables S27–30) and serum chemistry parameters (Tables S31–35). However, significant changes in specific parameters were noted infrequently. For example, Hb was significantly lower relative to the control group (154.00 ± 12.46 g/L; *p* < 0.05, Table S29) during study week 13 for the 60 mg/kg.bw (139.00 ± 4.69 g/L) and 120 mg/kg.bw treatment groups (140.33 ± 2.66 g/L). Additionally, the RDW value of the 120 mg/kg.bw group (16.22 ± 0.41%) was markedly lower than that of the 60 mg/kg.bw group (17.18 ± 0.82%) during week 13 (*p* < 0.01). In terms of serum chemistry, only ALB showed significant differences during week 13 (*p* < 0.01, Table S31), i.e., 60 mg/kg.bw group (29.42 ± 1.38 g/L) and 120 mg/kg.bw group (28.90 ± 1.05 g/L) versus control (31.60 ± 0.81 g/L). The majority of hematological and biochemical indices were significantly increased or decreased, compared with values before treatment (*p* < 0.05/0.01). The changes observed between pre- and post-dosing may be related to the feeding environment and tended to be stable after acclimation for 2 weeks. Moreover, differences were minimal with no evidence of dose- and time-response relationships, and were therefore not attributed to treatment with TA.

### Organ Coefficients

The organ-to-weight ratios of experimental dogs are presented in Table S36. The 20 mg/kg.bw treatment group displayed increased uterine and ovarian coefficients relative to the other three groups at week 13 of treatment (*p* < 0.01), i.e., 20 mg/kg.bw group, 0.21 ± 0.06% and 0.02 ± 0.0003% versus control, 0.04 ± 0.004% and 0.010 ± 0.003%; 60 mg/kg.bw group, 0.01 ± 0.002% and 0.005 ± 0.0006%; 120 mg/kg.bw group, 0.03 ± 0.02% and 0.008 ± 0.004%, respectively. Histopathological examination disclosed that two animals from the 20 mg/kg.bw group were in the menstrual cycle, and therefore, the increased values may have been related to the physiological cycle with no correlation to TA administration.

### Histopathological Changes

Gross necropsy findings demonstrated no adverse effects on organs after 13 weeks of TA treatment. As shown in Table 2 and Fig. S2, histopathological findings showed deposition of bluish-purple calcium salts in the individual collecting tubules near the renal medulla and nipple from two female dogs in the control and 20 mg/kg.bw groups (Fig. S2A) respectively. Furthermore, interstitial inflammation of prostate was observed in one male dog from the control and three TA groups. However, these lesions were mild and comparable among groups, and no similar changes were observed during the recovery period (Fig. S2A), and therefore considered unrelated to TA treatment effects. Interstitial pneumonia of varying severity was observed in all groups at the end of the administration (Fig. S2A) and recovery periods (Fig. S2B). Moreover, livers of a number of animals characteristically displayed spot-like necrosis or foci of scattered hepatocytes or microscopic granulomas. However, these lesions were mild and similar among groups and no dose dependency was observed (Fig. S2A and S2B), suggesting no association with TA treatment effects. In addition, two female dogs from the 20 mg/kg.bw group showed thickened endometrium, swollen glands, secretions in the gland cavity, and multiple large mature luteas in the ovary (Fig. S2C) at the end of week 13, which were attributable to the menstrual cycle and unrelated to TA administration.

**Table 2:** Summary of histopathological findings

Time	Organ	Pathologic changes	Grading	Control		TA (mg/kg.bw)					
						20		40		60	
				♂	♀	♂	♀	♂	♀	♂	♀
Week 13	Kidney	Calcium salt deposits	±	0	1	0	1	0	0	0	0
			+	0	1	0	1	0	0	0	0
	Lung	Interstitial pneumonia	+	2	1	2	2	1	1	2	2
			++	0	1	0	0	1	1	0	0
	Liver	Scattered spotty necrosis or small focal inflammation	±	1	2	1	1	1	1	1	1
			+	0	0	1	0	0	0	0	0
		Micro-granulomas	±	1	1	0	0	1	0	1	0
		Focal necrosis	±	0	0	0	0	0	0	1	0
	Prostate	Interstitial inflammation and acinar dilatation	+	1	–	1	–	1	–	1	–
	Uterus	Thickened endometrium hyperplasia, increased glands, and secretions	+	–	0	–	2	–	0	–	
	Ovary	Mature corpus luteum	+	–	0	–	2	–	0	–	0
Week 17	Lung	Interstitial pneumonia	+	1	1	1	1	1	1	1	1
	Liver	Scattered spotty necrosis or small focal inflammation	±	0	0	0	1	1	1	0	0
		Micro-granulomas	±	0	1	0	0	0	0	0	1

The numbers of single sex animals subjected to histopathological examination in each group during the administration and recovery periods are two and one, respectively

Grading, ±: suspicious or occasional minimal, +: mild or minor, ++: moderate or numerous

Values represent the number of animals with findings

## Discussion

Natural drugs are increasingly popular in healthcare and other areas worldwide. However, safety remains a major public health problem, since these agents are generally perceived as being harmless to the body and often used for self-therapy without supervision. Although bioactive compounds in medicinal plants clearly exert a variety of beneficial biological effects in humans, knowledge of their potential toxicities is limited. Leaves of *A. scholaris* have traditionally been used to treat whooping cough in Dai people from Yunnan Province of China. Previously, our group demonstrated the efficacy of total indole alkaloid extract of *A. scholaris* in respiratory management. Considering its potential value, absence of a robust toxicological study and diversity of experimental animals, further evidence of the pharmacology and safety of highly concentrated indole alkaloids of leaves is necessary prior to clinical use. Therefore, a comprehensive preclinical program including both acute and sub-chronic studies was conducted in higher-level hierarchical classification animals frequently used for pharmaceutical safety evaluation such as dogs, although the safety of stem bark of *A. scholaris* have been evaluated individually [36]. Accordingly, the present study focused on acute and sub-chronic oral toxicity of indole alkaloids of leaves from *A. scholaris* in dogs.

The acute toxicity test is effectively used for evaluating adverse effects appearing within a short time after administration of a single large dose of the test substance or multiple doses within 24 h. In our experiments, the lethal dose of TA was not determined in the acute oral toxicity study. Instead, TA was administered orally to beagle dogs (1 male and 1 female) at a dose of 4 g/kg.bw, which was shown to induce unsteady gait, drooling, emesis, and perioral mucosa reddening in both genders. These symptoms may be related to the central nervous system and autonomic system, which requires further investigation in clinical trials. The results indicate no significant toxicity, suggesting that TA is well tolerated by experimental non-rodents.

Thirteen-week sub-chronic toxicity studies are extensively used for evaluating long-term toxicological effects and safety profiles of drugs [37]. The current doses were selected based on the results of a chronic toxicity study on rats in which oral administration of TA (50, 100, or 300 mg/kg.bw) had no significant toxicity symptoms [33] and the pharmacodynamics dose in rats was 15 mg/kg.bw [25]. Another important reason for our dose selection was because a 2 mg/kg.bw dose was suggested for potential TA applications in humans [32], which guided us to limit the highest dose to 120 mg/kg.bw. No mortality or morbidity was observed during the course of the study. However, reversible and test substance-related emesis, loose feces and drooling was detected in dogs of both genders treated with TA at doses of 20, 60, 120 mg/kg.bw. These clinical signs were not observed during the recovery period. Our findings were not considered toxic effects of the test material, as neither histopathological changes in the gastrointestinal tract nor alterations in body weight were observed. Emesis may be attributable to the fact that dogs are sensitive to the gag reflex and prone to vomiting. Additionally, infrequent green viscous material in feces was observed in one male and one female treated with 120 mg/kg.bw TA, which could be due to TA-stained ingesta.

The hematopoietic system, which is highly sensitive to toxic substances, undergoes alterations upon ingestion of poisonous plants [38, 39], and is therefore an important indicator of physiological and pathological conditions in humans and animals [40, 41]. We assayed full sets of hematological and blood biochemistry parameters for each group at five time-points (acclimation, weeks 5, 9 and 13 after treatment and end of the recovery period). No significant TA-related effects were observed on any of the hematology or clinical chemistry values, except reduced RDW, which was markedly lower in the 120 mg/kg.bw than 60 mg/kg.bw group at week 13. The Hb content was substantially lower than the control group in the 60 and 120 mg/kg.bw groups at week 13. In addition, ALB values in the 60 and 120 mg/kg.bw groups at week 13 were reduced relative to the control group. However, these data fluctuated within the historical range and exhibited no dose- or time-response relationships with physiological meaning. Small but significant changes in these parameters were observed in TA-treated dogs. However, with no changes in associated clinical signs or pathological markers and no dose–response or time-response relationships, these observations cannot be considered adverse effects with biological significance. Electrocardiography and ophthalmologic analyses revealed no significant differences among groups. Data from urine and fecal analyses additionally showed no test substance-related changes in both genders among the main treatment, recovery and vehicle control groups. Furthermore, no test article-related differences were evident in rectal temperature, bone marrow, macroscopic or microscopic evaluations.

Absolute and relative organ weights in all treatment groups were not significantly different from those of the control group, except that ovarian and uterine coefficients were markedly higher in the 20 mg/kg.bw group than those of the other three groups at week 13. However, pathological examination showed thickened endometrium, swollen glands, and secretions in the gland cavity as well as multiple large mature ca in the ovary, indicative of the secretory phase of menstrual cycle. Accordingly, these changes were not considered to be of TA-related toxicological significance. Gross or microscopic pathological examination of organs and tissues disclosed similar findings for all groups, with changes detected during the course of treatment or after recovery.

Minor abnormalities of kidney, lung, liver, prostate, uterus and ovary observed in TA-administered dogs were incidental with no dose correlations and/or a similar degree or type as the control group. These spontaneously detected changes were not associated with other clinical or necropsy observations. Control and TA groups displayed no significant differences in type, prevalence or severity of deviations. Accordingly, the changes were deemed incidental with minimal toxicological consequences for dogs treated with oral doses of 20, 60 and 120 mg/kg.bw TA. Notably, the incidence and severity of interstitial pneumonia were similar between the TA administration and control groups, consistent with a previous report that reflux after gavage dosing or accidental administration of test formulation led to higher frequency of pneumonia in dogs [42]. Lesions of other organs showed no dose- or time-dependent relationships and were not physiologically significant.

Overall, data from clinical observations and laboratory indices suggest no significant treatment-related changes. Accordingly, the maximum tolerance dose (MTD) and the no-observed-adverse-effect-level (NOAEL) for TA extract from leaves of *A. scholaris* was estimated as 4 g/kg.bw and 120 mg/kg.bw in acute and sub-chronic toxicity studies of beagle dogs, respectively.

## Conclusions

A summary of relevant findings from an acute oral toxicity study as well as a 13-week oral administration and 4-week recovery period in beagle dogs is presented. The overall weight of evidence regarding TA safety in dogs based on current as well as previously published data clearly suggests a high degree of tolerability. For potential application of TA in humans, a 2 mg/kg.bw dose is suggested, which is lower than NOAEL values of 4 g/kg.bw and 120 mg/kg.bw obtained from the acute and sub-chronic studies, respectively. Given the potential health benefits of oral capsules formulated with TA and data presented supporting its safe usage in dogs, we propose that TA may be a safe and effective botanical drug for use in humans.

## Materials and Methods

### Plant Material

Leaves of *A. scholaris* were purchased from Datang-Hanfang Medicine Co. Ltd. (Pu’er, China), and identified by Dr. Xiao-Dong Luo, Kunming Institute of Botany, Chinese Academy of Sciences. Plant names were checked using the website https://www.theplantlist.org. A voucher specimen (No. Luo20060407) has been deposited in State Key Laboratory of Phytochemistry and Plant Resources in West China, Chinese Academy of Sciences.

### Alkaloid Preparation

Dried and powdered leaves of *A. scholaris* were extracted with 90% EtOH under reflux conditions (3 h × 4) and the solvent evaporated *in vacuo* to obtain ethanolic extract. The ethanolic extract was dissolved in 0.3% aqueous HCl solution, filtered, and the residue used as the non-alkaloid fraction. The acidic solution, adjusted to pH 9–10 with 10% aqueous ammonia, was extracted with EtOAc to obtain the TA fraction (batch number, 20070512). HPLC/UV was applied to determine the four major alkaloids of TA (supporting information, S2.1.–2.2.). Typical HPLC/UV chromatograms of TA are presented in Fig. S1B. The chromatography profile displayed four peaks with retention times of 7.464 (19-epischolaricine), 7.965 (scholaricine) 12.810 (vallesamine) and 21.874 min (picrinine). The purities are all more than 90% and the contents are 19-epischolaricine (2%), scholaricine (6%), vallesamine (6%), picrinine (10%) within the expected range, respectively.

### Experimental Animals

Thirteen (13) male and 13 female purebred beagle dogs aged 6–10 months (body weights, 7–10 kg) in good health were provided by Guangzhou Pharmaceutical Industry Research Institute (Guangzhou, China). All dogs were inoculated with the relevant vaccines and tested for parasites, hematological and serum biochemical parameters prior to arrival at our laboratory. Dogs were housed (one per stainless steel cage) in a room at a temperature range of 21–26 °C and relative humidity of 40–70% under a 12 h light–dark cycle and air ventilation rated of 9 times/hour. Food and water were freely available. Three or four weeks of quarantine were required before TA administration. Our experiments were reviewed and approved by the Institutional Animal Care and Use Committee of the Yunnan Institute of Medical Material. All animals were fasted but had access water for 10–12 h before initiation of the experiment.

### Acute Toxicity Study

The acute toxicity protocol in dogs was designed in accordance with the Testing Guidelines for safety evaluation of drugs (Notification [Z] GPT2-1) issued by China Food and Drug Administration in March 2005 [43]. We selected one female and one male purebred dog for TA treatment, which was administered via capsules at a dose of 4 g/kg.bw. This value was determined from our preliminary experiments on dogs in which oral administration of TA (1 and 4 g/kg.bw) via capsules had no obvious effects on survivability, macroscopic or microscopic parameters. The required amount of test article for each animal was weighed and placed into the appropriate number of tared gelatin capsules (size 12 capsules manufactured by Yunnan Guangdeli Capsule co. Ltd, accommodating up to 1.5 g/capsule). To facilitate smooth swallowing, capsules were suspended in water and pumped along the dog's upper throat, and then the mouths of animals were closed for a few seconds.

### Sub-chronic Toxicity Study

To assess the target organs and reversibility of *A. scholaris,* a 13-week administration period, followed by 4 weeks of recovery, was conducted on dogs according to the Testing Guidelines for Safety Evaluation of Drugs (Notification [Z] GPT3-1) issued by China Food and Drug Administration in March 2005 [44]. Dose levels were selected based on the results of a chronic toxicity study on rats in which oral administration of TA (50, 100, or 300 mg/kg.bw) had no significant influence on survivability, macroscopic and microscopic parameters [33] and the pharmacodynamics dose in rats was 15 mg/kg.bw [25]. In addition, a 2 mg/kg.bw dose was suggested for potential TA applications in humans [32]. In total, 12 female and 12 male purebred dogs were allocated into 4 groups, each containing 6 dogs (3 female and 3 male), for administration of TA capsules at doses of 0 (control), 20, 60, 120 mg/kg.bw. Gelatin capsules filled with TA extract were orally administered to dogs. After 13 weeks of treatment, 4 dogs (2 males and 2 females) of each group were sacrificed. The remaining animals were continuously observed over the 4-week recovery phase and sacrificed after the experimental period. Animals in the control group received empty capsules equivalent to that used for the same sex in the 120 mg/kg.bw treatment group. The required amount of test article for each animal was weighed and placed into the appropriate number of tared gelatin capsules (size 12 capsules manufactured by Yunnan Guangdeli Capsule co. Ltd, accommodating up to 1.5 g/capsule).

### Evaluated Parameters

#### Clinical Observations

All the indices determined and frequencies are shown in Table 3, For the acute toxicity study, appearance and behavioral activities were supervised systematically after a single oral TA administration, including abnormal appearance, behavior, activity level, pupil size, appetite, food consumption, mental state, urination and defecation behavior, skin and hair, nausea, emesis, chatter, and ataxia. Body weights were evaluated during acclimation (study days − 21 and − 1), day of administration (1 day) and recovery observation (days 7 and 14), and rectal temperatures measured at 0, 30, 60, 120 min, 1, 7 and 14 days with an anal thermometer. The number of living dogs was recorded after 24 h and once-daily examinations conducted for a further 14 days.

**Table 3:** Determination of indices and frequencies

Items	Acute toxicity					Sub-chronic toxicity					
	− 21 day	− 1 day	1 day	7 day	14 day	− 2 week	− 1 week	5 week	9 week	13 week	17 week
General symptoms	×	×	×	×	×	Once daily					
Food consumption	×	×	×	×	×	×	×	Once weekly			
Body weight	√	√	√	√	√	√	√	Once weekly			
Temperature	√	√	√	√	√	√	√	√	√	√	√
	Before exposure and 30, 60 and 120 min post-exposure										
Electrocardiography	√	√	√	√	√	√	√	√	√	√	√
	30, 60, and 120 min post-exposure										
Ophthalmological examination	×	×	×	×	×	√	×	×	×	√	√
Fecal analysis	√	√	√	√	√	√	×	×	×	√	√
Urinalysis	√	√	√	√	√	√	√	√	√	√	√
Hematology	√	√	√	√	√	√	√	√	√	√	√
Serum chemistry	√	√	√	√	√	√	√	√	√	√	√
Bone marrow	×	×	×	×	×	×	×	×	×	√	√
Gross observation and necropsy	×	×	×	×	×	×	×	×	×	√	√
Histopathology	×	×	×	×	×	×	×	×	×	√	√

“√”: The item was evaluated at the planned time-point

“×”: The item was not evaluated

For the sub-chronic toxicity study, mortality and clinical signs were evaluated daily from the beginning of the quarantine period to the end of the experiment. Each animal was examined at least once daily for changes in behavior and reaction to treatment or illness. Observations included, but were not limited to: changes in skin and fur, eyes and mucous membranes, respiratory, circulatory, autonomic, and central nervous systems, and behavior patterns. Body weights were examined weekly and food consumption assessed daily and subjected to statistical analyses weekly during the study and recovery periods. Rectal temperature was measured on weeks − 2, − 1, 5, 9, 13 and 17 with an anal thermometer. Ophthalmological examinations were performed on weeks − 2, 13 and 17.

#### Clinical Parameters

The following indices (electrocardiography, hematology, serum chemistry and urinalysis) were examined during acclimation (study weeks − 2 and − 1), day of administration (day 1), and recovery observation (days 7 and 14) in the acute toxicology study and during acclimation (study weeks − 2 and − 1),study weeks 5 and 9, and scheduled necropsy (weeks 13 and 17) in the sub-chronic study.

For urinalysis, urine samples were evaluated with an autoanalyzer (SIEMENS ClinitekStatus, German Micro AUTION MA-4260, Arkray Global Business, Inc., Kyoto, Japan). The indices examined included specific gravity (SG), pH, leukocyte count (WBC), protein (PRO), glucose (GLU), ketones (KET), urobilinogen (URO), bilirubin (BIL) and occult blood (BLO).

For fecal analysis, occult blood was measured using test paper (Zhuhai Beso Biotechnology co. Ltd, Zhejiang, China). Microscopic examination of parasitic eggs was additionally conducted. Fecal analysis in the acute toxicity test during acclimation (days − 21 and − 1), day of administration (day 1) and recovery observation (days 7 and 14) and in the sub-chronic toxicity test was performed during acclimation (week − 2) and scheduled necropsy (study weeks 13 and 17).

In the lead electrocardiographic (ECG) test, data on P wave, R wave, T wave, P-R interval, Q-T interval, QRS duration, S-T segment and heart rate were collected using the RM6240C physiological signal collection system (Beijing Jinyang Wanda Technology Co. Ltd, Beijing, China) with the II limb lead method. ECG evaluation of acute toxicity was performed at 30, 60 and 120 min after TA intervention and the scheduled time shown in Table 3.

*Hematology:* Blood samples were collected in ethylenediaminetetraacetic acid (EDTA)-containing tubes using a hematology autoanalyzer (HEMAVET 950, Drew Scientific, USA) for measurement of white blood cell (WBC), neutrophil (NE), lymphocyte (LY), monocyte (MO) and red blood cell (RBC) counts, hemoglobin (Hb), red blood cell distribution width (RDW), mean corpuscular volume (MCV), mean corpuscular hemoglobin (MCH), mean corpuscular hemoglobin concentration (MCHC), reticulocyte (RET), and platelet (PLT) levels. Blood samples (1.8 mL) were mixed with 0.2 mL sodium citrate for measurement of prothrombin time (PT) using a blood coagulation analyzer (C2000-4, Beijing Precil Instrument Co., Ltd., Beijing, China).

##### Serum chemistry

Blood samples were centrifuged for 10 min at a speed of 3000 rpm in vacuum blood collection tubes and collected in 1.5 mL centrifuge tubes for measuring alanine aminotransferase (ALT), aspartate aminotransferase (AST), total protein (TP), albumin (ALB), total bilirubin (TBil), alkaline phosphatase (ALP), blood urea nitrogen (Bun), creatinine (Cre), glucose (GLU), triglyceride (TG), total cholesterol (TC), sodium ion (Na^+^), potassium ion (K^+^), chloride ion (Cl^−^), ionized calcium (iCa), total calcium (TCa) and potential of hydrogen (pH) values using the Beckman AU680 automatic biochemistry analyzer (Kraemer Boulevard, Brea, USA).

##### Bone Marrow Examination

It was performed after 13 weeks of drug administration and a 4-week recovery period. A small amount of bone marrow extruding from sternal stems of sacrificial animals were mixed with serum and smeared uniformly, dried, stained with Wright’s stain (Leagene Biotechnology Co., Ltd., Beijing, China), and examined via microscopy. The test parameters included the proportions of granulocytes, erythrocytes, lymphocytes, monocytes and ratio of plasmocytes. No bone marrow examinations were performed for the acute toxicity test.

##### Histopathology

For the sub-chronic toxicity test, weights of individual organs isolated from each animal following sacrifice, including spleen, liver, thymus, heart, lung, brain, kidneys, adrenal glands, ovaries, uterus, testes and epididymis, were examined. The following tissues were isolated immediately after euthanasia: abnormal lesions, skin, mammary gland, spleen, pancreas, jejunum, stomach, duodenum, ileum, cecum, colon, mesenteric lymph node, salivary gland, submandibular lymph node, liver, sternum, thymus, heart, lung, trachea, esophagus, thyroid along with parathyroid, tongue, aorta, sciatic nerve, skeletal muscle, femur, brain, pituitary gland, eyes, kidneys, adrenal glands, urinary bladder, ovaries, uterus, prostate, testes and epididymis. All tissues were fixed with 10% neutral buffered formalin. Organs and tissues were routinely processed, embedded in paraffin, sectioned at 4 μm thickness, and stained with hematoxylin–eosin for histopathological examination.

### Statistical Analysis

Data are presented as means ± standard deviation (SD). Statistical evaluation was performed using SPSS 17.0 software (Chicago, IL, USA) and variations in data for all parameters assessed for homogeneity via Bartlett’s procedure. In cases where data were homogeneous, one-way analysis of variance for homogeneity (ANOVA) was used, while in heterogeneous cases, the Kruskal–Wallis test was applied. When significant differences were indicated, least significant difference (LSD) and Dunnett’s T3 multiple tests were employed for comparison of control and treated groups. The nonparametric test was selected for ranked data. Results were considered significant at *p* values < 0.05.

## Electronic supplementary material

Below is the link to the electronic supplementary material.

Supplementary file1 (PDF 3491 kb)

## Abbreviations

TA: Total alkaloids extract
H&E: Hematoxylin–Eosin
WBC: White blood cell count
NE: Neutrophils
LY: Lymphocytes
MO: Monocytes
RBC: Red blood cell count
Hb: Hemoglobin
RDW: Red blood cell distribution width
MCV: Mean corpuscular volume
MCH: Mean corpuscular hemoglobin
MCHC: Mean corpuscular hemoglobin concentration
PLT: Platelets
PT: Prothrombin time
RET: Reticulocytes
ALT: Alanine aminotransferase
AST: Aspartate aminotransferase
TP: Total protein
ALB: Albumin
TBIL: Total bilirubin
ALP: Alkaline phosphatase
BUN: Blood urea nitrogen
CRE: Creatinine
GLU: Glucose
TG: Triglycerides
TC: Total cholesterol
CK: Creatine kinase
K^+^: Potassium ions
Na^+^: Sodium ions
Cl^−^: Chloride ions
iCa: Ionized calcium
TCa: Total calcium
pH: Potential of hydrogen
PRO: Protein
KET: Ketone body
BLO: Urine occult blood
BIL: Bilirubin
OB: Fecal occult blood
