# Supplementary material for: Acute and Sub-chronic Toxicity of Indole Alkaloids Extract from Leaves of Alstonia scholaris (L.) R. Br. in Beagle Dogs
Source: Nat Prod Bioprospect. 2020 Jun 10;10(4):209–20. doi: 10.1007/s13659-020-00246-0 (PMC7367998; doi:10.1007/s13659-020-00246-0)
Supplement: Supplementary file 1 — Supplementary file1 (PDF 3491 kb) [file 13659_2020_246_MOESM1_ESM.pdf]

Supporting Information for

Acute and Sub-chronic toxicity of indole alkaloids extract from leaves of *Alstonia scholaris* (L.) R. Br. in beagle dogs

Yun-Li Zhao [a,c,1], Min Su [b,1], Jian-Hua Shang [b,c,1], Xia Wang [b], Guang-Lei Bao [b], Jia Ma [b], Qing-Di Sun [d], Fang Yuan [b], Jing-Kun Wang [b,*], Xiao-Dong Luo [a,c,*]

[a] *State Key Laboratory of Phytochemistry and Plant Resources in West China, Kunming Institute of Botany, Chinese Academy of Sciences, Kunming 650201, P. R. China*

[b] *Yunnan Institute of Medical Material, Kunming 650111, P. R. China*

[c] *Key Laboratory of Medicinal Chemistry for Natural Resource, Ministry of Education and Yunnan Province, School of Chemical Science and Technology, Yunnan University, Kunming 650091, People's Republic of China*

[d] *Jiangsu Nhwa Pharmaceutical Co., Ltd, Xuzhou, 221009, P. R. China*

---

* Corresponding author. Tel.: +86 871 65223177; fax: +86 871 65220227.

E-mail address: xdluo@mail.kib.ac.cn (X.-D. Luo); wjkyimm@163.com (J.-K. Wang)

[1]These authors contributed equally.

Contents

S1. The “hot off the press” compounds and total synthesized *Alstonia* alkaloids 4

Table S1 Eight “hot off the press” compounds as specified in *Natural Products Report* 4

Table S2 Sixteen total synthesized Alstonia alkaloids by chemists 4

S2. Alkaloids quantitative analysis 4

S2.1. Sample preparation 4

S2.2. HPLC/UV quantitative analysis 5

Fig. S1. HPLC/UV chromatograms of total alkaloids (285 nm) 6

S3. The results of acute toxicity on beagle dogs 7

Table S3 Bodyweight after single oral administration of TA 7

Table S4 Rectal temperature after single oral administration of TA 7

Table S5 Hematology results after single oral administration of TA 7

Table S6 Biochemical indices after single oral administration of TA 8

Table S7 Serum electrolytes and coagulation indicators after single oral administration of TA 8

Table S8 ECG results after single oral administration of TA (No.: 62244M, 1#) 9

Table S9 ECG results after single oral administration of TA (No.: 71192F, 20#) 9

Table S10 The urine and feces results after single oral administration of TA 9

S4. The results of sub-chronic toxicity on beagle dogs 10

Table S11 Food intake after treatment with TA for 13 weeks via group comparison 10

Table S12 Body weights after TA treatment for 13 weeks via group- and self- comparison 11

Table S13 Temperatures after TA treatment for 13 weeks via group- and self- comparison 12

Table S14 Ophthalmological examination before treatment with TA 13

Table S15 Ophthalmological examination after treatment with TA for 13 weeks 14

Table S16 Ophthalmological examination after withdrawal TA for 4 weeks 15

Table S17 Urinalysis before the administration of TA for -1 week 16

Table S18 Urinalysis before the administration of TA for -2 week 18

Table S19 Urinalysis after the administration of TA for 5 weeks 19

Table S20 Urinalysis after the administration of TA for 9 weeks 20

Table S21 Urinalysis after the administration of TA for 13 weeks 21
Table S22 Urinalysis after the withdrawal of TA for 4 weeks 22
Table S23 SG, PH, and URO results during the study period via group- and self-comparison 23
Table S24 Feces examination during the study period via group- and self-comparison 24
Table S25 Electrocardiographic examination during the study period via group comparison 25
Table S26 The results of bone marrow examination at different times via group comparison 26
Table S27 Hematology results before and after TA administration via group comparison 27
Table S28 Hematology results after TA withdrawal for 4 weeks via group comparison 28
Table S29 Hematology results before and after TA administration via self-comparison 29
Table S30 Hematology results after TA withdrawal via self-comparison 30
Table S31 Biochemistry results after TA administration via group comparison 31
Table S32 Biochemistry results after TA withdrawal for 4 weeks via group comparison 32
Table S33 Biochemistry results after TA administration via self-comparison 33
Table S34 Biochemistry results after TA withdrawal for 4 weeks via self-comparison 34
Table S35 Electrolytes results during the study period via group- and self- comparison 35
Table S36 Organ coefficients during the study period via group comparison 36
Fig. S2A. Representative sections of dogs in respect of 13 weeks treatment demonstrating histopathology . 37
Fig. S2B. Representative sections of dogs in the recovery period demonstrating histopathology 39
Fig. S2C. Representative uterus and ovary sections of two female dogs in the 20 mg/kg.bw at week 13 40

S1. The “hot off the press” compounds and total synthesized *Alstonia* alkaloids

Table S1 Eight “hot off the press” compounds as specified in *Natural Products Report*

No.	Compounds	No.	Compounds
1	E-alstoscholarines [1]	5	alstolactine A [2]
2	Z-alstoscholarines [1]	6	alstoscholarisine A [3]
3	scholarisine A [4]	7	alstoscholarisines F [5]
4	alstrostine A [6]	8	alstoscholarisines H [7]

Table S2 Sixteen total synthesized Alstonia alkaloids by chemists

No.	Compounds	No.	Compounds
1	scholaricine A [8-11],	9	scholaricine K [12]
2	E-alstoscholarine [13]	10	alstolactine A [12]
3	Z-alstoscholarine [13]	11	alstoscholaricine B [14]
4	alstoscholaricine A [15, 16]	12	alstoscholaricine C [14]
5	alstoscholaricine H [7]	13	alstoscholaricine D [14]
6	scholaricine G [17-20]	14	alstoscholaricine E [14]
7	scholaricine K [12]	15	picrinine [21, 22]
8	alstolactine A [12]	16	strictamine [16, 23]

S2. Alkaloids quantitative analysis

S2.1. Sample preparation

The sample of total alkaloids was pulverized into fine powder. Then 1.0 mg of the total alkaloids was

accurately weighed and extracted with 1.0 mL of 50% methanol in ultrasonic water bath for 30 min at 25 °C. The sample solution was filtered through a 0.22 μm membrane before use. A 10 μL-aliquot was injected for UHPLC/UV quantitative analysis.

S2.2. HPLC/UV quantitative analysis

The quantitative analysis was performed on an a Agilent 1290 series HPLC system (Agilent Technologies, Germany) comprised a quaternary pump, an auto-sampler, a column temperature controller and a PDA detector. All samples were separated on an ACQUITY UPLC® CSH column (2.1 × 100 mm, 1.7 μm) equipped with a Waters VanGuard pre-column (5 mm × 2.1 mm I.D., 1.8 μm). The mobile phase consisted of acetonitrile (A) and water containing 0.5% trifluoroacetic acid (v/v, B) at the ratio of 18 : 82. The following linear elution gradient was used: 0-10 min, 4-16% A; 10-15 min, 16-20% A; 15-18 min, 20% A; 18-20min, 20-95% A. The flow rate was 0.20 mL/min. The column temperature was maintained at 45 °C. The sample tray temperature was maintained at 25 °C. The detection wavelength was set from 190 to 600 nm, and the samples were detected at 285 nm in accordance with the maximum absorption of the analytes. Typical HPLC/UV chromatograms of TA are presented in Fig. S1. As shown in Fig. S1B, the chromatography profile displayed four peaks with retention times of 7.464 (19-epischolaricine), 7.965 (scholaricine) 12.810 (vallesamine) and 21.874 minutes (picrinine). The purities are all more than 90% and the content is 19-epischolaricine (2%), scholaricine (6%), vallesamine (6%), picrinine (10%) within the expected range, respectively.

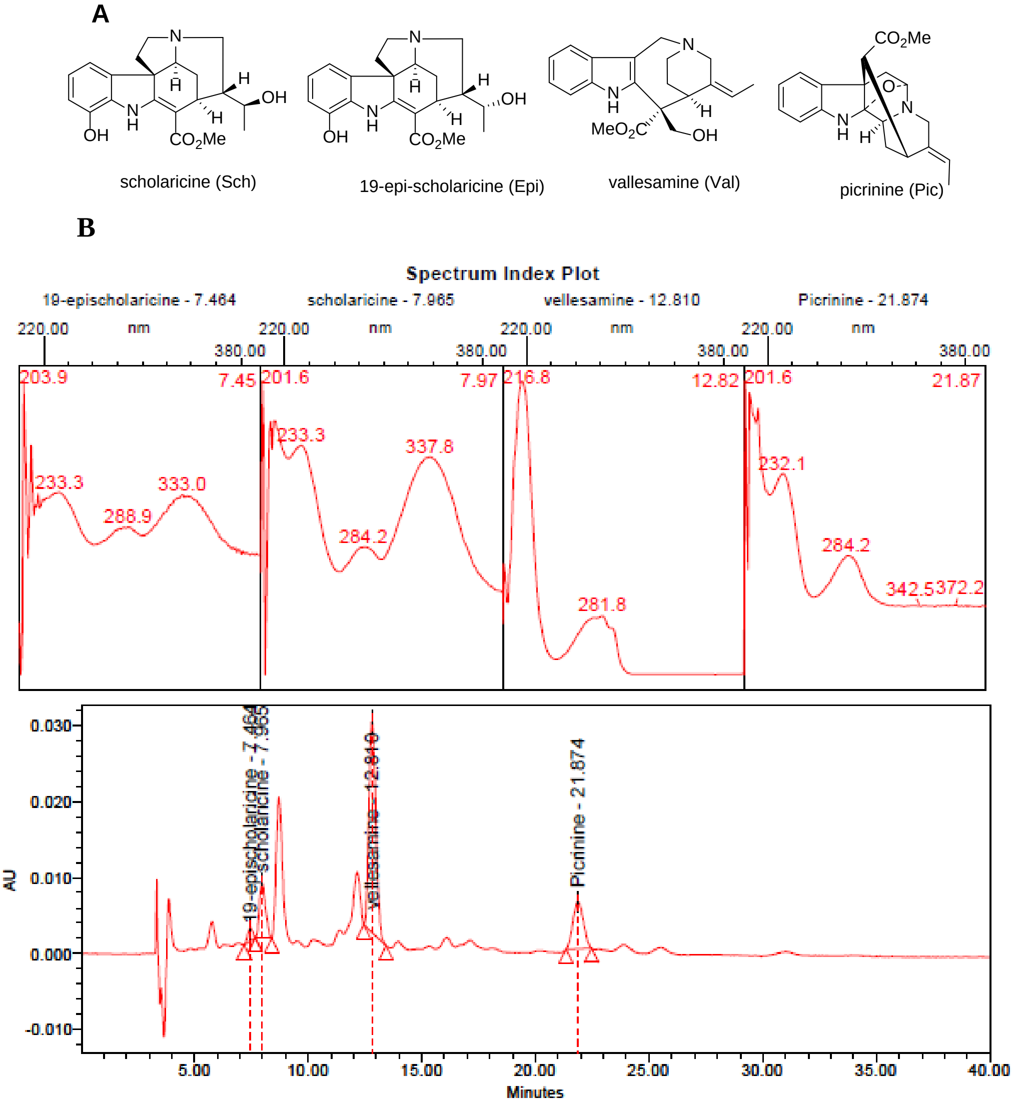

Fig. S1. HPLC/UV chromatograms of total alkaloids (285 nm)

A. Four major alkaloids from leaf of *A. scholaris.*

B. HPLC/UV chromatograms of total alkaloids.

S3. The results of acute toxicity on beagle dogs

Table S3 Bodyweight after single oral administration of TA

表 9　灯台叶碱提取物 Beagle 犬灌胃给药急性毒性试验体重（kg）

动物编号	笼号	剂量（g/kg）	性别	给药前	给药后		
					第 1 天	第 7 天	第 14 天
62244M	1	4.0	♂	9.03	9.12	8.96	8.90
71192F	20	4.0	♀	9.34	9.19	9.04	9.14

Table S4 Rectal temperature after single oral administration of TA

表 10　灯台叶碱提取物 Beagle 犬灌胃给药急性毒性试验体温（℃）

动物编号	笼号	性别	给药前		给药后					
			第 1 次	第 2 次	30 min	60 min	120 min	第 1 天	第 7 天	第 14 天
62244M	1	♂	38.7	38.6	38.9	39.0	38.5	38.6	38.7	39.0
71192F	20	♀	38.8	37.9	38.8	39.1	38.5	38.3	38.3	38.6

Table S5 Hematology results after single oral administration of TA

表 11　灯台叶碱提取物 Beagle 犬灌胃给药急性毒性试验血液学检测结果

项目	单位	62244M(1#)					71192F(20#)				
		给药前		给药后			给药前		给药后		
		第 1 次	第 2 次	第 1 天	第 7 天	第 14 天	第 1 次	第 2 次	第 1 天	第 7 天	第 14 天
WBC	K/uL	10.30	6.94	9.18	7.42	7.12	5.66	5.86	5.98	5.62	5.90
NE#	K/uL	5.64	4.39	5.97	4.98	5.12	3.93	3.52	3.78	4.23	4.36
LY#	K/uL	3.48	0.89	1.90	1.75	1.19	1.03	0.76	0.85	0.91	0.78
MO#	K/uL	0.40	0.30	0.47	0.55	0.70	0.32	0.32	0.30	0.38	0.67
EO#	K/uL	0.74	1.32	0.78	0.13	0.11	0.33	1.19	1.00	0.09	0.09
BA#	K/uL	0.03	0.05	0.05	0.01	0.01	0.05	0.07	0.05	0.01	0.01
NE%	%	54.78	63.20	65.01	67.14	71.93	69.51	60.08	63.15	75.27	73.86
LY%	%	33.8	12.79	20.86	23.59	16.70	18.15	12.99	14.16	16.14	13.15
MO%	%	3.89	4.29	5.13	7.41	9.77	5.69	5.38	5.08	6.79	11.29
EO%	%	7.23	18.98	8.49	1.75	1.51	5.77	20.36	16.71	1.65	1.53
BA%	%	0.30	0.75	0.52	0.12	0.10	0.87	1.19	0.90	0.15	0.16
RBC	M/uL	8.28	7.82	8.30	8.36	8.09	7.16	7.62	8.03	7.55	7.73
Hb	gL	16.9	16.8	19.0	16.5	16.6	14.6	16.8	19.2	15.3	16.1
HCT	%	54.6	50.3	54.4	55.1	53.7	48.8	51.1	54.2	51.0	52.6
MCV	fL	65.9	64.3	65.5	65.9	66.4	68.1	67.0	67.5	67.6	68.1
MCH	Pg	20.4	21.5	22.9	19.7	20.5	20.4	22.0	23.9	20.3	20.8
MCHC	gL	31.0	33.4	34.9	29.9	30.9	29.9	32.9	35.4	30.0	30.6
RDW	%	13.8	13.8	13.9	14.4	14.0	14.4	13.8	14.2	14.2	14.2
PLT	K/uL	277	274.0	368.0	274.0	314.0	220.0	342.0	415.0	235.0	263.0
MPV	fL	13.0	11.0	10.8	14.5	13.5	13.8	10.9	9.3	15.1	13.1

Table S6 Biochemical indices after single oral administration of TA

表 12 灯台叶碱提取物 Beagle 犬灌胃给药急性毒性试验血清生化检测结果

项目	单位	62244M(1#)					71192F(20#)				
		给药前		给药后			给药前		给药后		
		第 1 次	第 2 次	第 1 天	第 7 天	第 14 天	第 1 次	第 2 次	第 1 天	第 7 天	第 14 天
ALT	U/L	34	25	24	32	23	18	18	29	21	31
AST	U/L	33	24	16	32	31	31	22	25	31	36
TG	mmol/L	0.22	0.24	0.17	0.33	0.71	0.69	0.33	0.20	0.73	0.54
TC	mmol/L	3.48	3.18	3.25	3.36	6.05	5.50	5.39	3.34	6.09	4.35
GLU	mmol/L	4.8	4.8	3.7	32.7	30.5	5.1	4.8	4.1	32.8	31.5
CRE	umol/L	114	109	153	208	179	123	110	169	184	196
Bun	mmol/L	3.5	3.1	2.8	2.8	4.5	4.1	4.5	3.1	4.1	3.2
ALP	U/L	85	87	92	96	101	111	104	96	116	98
CK	U/L	152	102	97	157	184	204	156	125	200	204
ALB	g/L	35.0	34.6	35.0	35.5	35.6	34.9	34.8	35.0	35.6	36.1
TP	g/L	76.8	8.4	83.2	102.4	113.3	83.4	64.5	89.9	114.9	105.4
GGT	U/L	4	4	6	0	2	2	5	5	-1	3
TBIL	umol/L	3.2	3.1	6.8	9.6	8.2	4.8	2.7	10.3	7.6	6.8

Table S7 Serum electrolytes and coagulation indicators after single oral administration of TA

表 13 灯台叶碱提取物 Beagle 犬灌胃给药急性毒性试验血清电解质和血凝检测结果

项目	单位	62244M(1#)					71192F(20#)				
		给药前		给药后			给药前		给药后		
		第 1 次	第 2 次	第 1 天	第 7 天	第 14 天	第 1 次	第 2 次	第 1 天	第 7 天	第 14 天
K	mmol/L	4.76	5.00	5.20	4.31	4.39	5.26	4.92	5.21	4.46	4.23
Na	mmol/L	151.7	145.1	146.5	134.7	138.5	144.6	145.7	147.8	130.5	140.1
Cl	mmol/L	111.6	121.4	121.0	101.9	112.5	114.2	116.9	114.8	99.2	106.1
iCa	mmol/L	1.35	1.35	1.23	1.33	1.30	1.44	1.40	1.29	1.27	1.38
TCa	mmol/L	2.63	2.64	2.41	2.60	2.53	2.81	2.73	2.52	2.48	2.69
PH	-	7.47	7.49	7.44	7.43	7.46	7.53	7.50	7.43	7.43	7.49
AB	mmol/L	18.7	21.8	19.8	21.0	20.9	16.4	17.8	22.1	19.8	22.5
AG	mmol/L	21.3	2.0	5.7	11.7	5.1	14.0	11.0	10.9	11.5	11.6
PT	sec	6.4	7.5	8.3	5.8	11.6	5.9	6.6	10.2	6.1	6.9
TT	sec	8.8	3.6	9.5	5.8	8.4	8.4	9.4	10.9	7.3	13.4
APTT	sec	16.1	17.8	35.9	57.5	18.8	15.3	17.5	34.1	32.0	19.7
FIB	g/l	2.94	3.13	2.77	1.51	1.55	2.85	3.23	1.93	1.79	2.89

Table S8 ECG results after single oral administration of TA (No.: 62244M, 1#)

表 14　灯台叶碱提取物 Beagle 犬灌胃给药急性毒性试验心电检查结果（编号 62244M(1#)）

时期		P 波时限(ms)	P 波振幅(mV)	R 波振幅(mV)	T 波振幅(mV)	ST 偏移量(mV)	P−R 间期(ms)	QRS 群间期(ms)	Q−T 间期(ms)	心率（次/分）
前期	第一次	55	0.15	1.01	0.08	0.01	85.33	98.00	329.00	131
	第二次	50	0.17	1.52	0.09	0.02	102.00	88.20	268.40	109
	给药前	45	0.16	1.73	0.08	-0.02	143.67	142.67	318.00	80
给药后	30min	40	0.33	1.37	0.38	0.06	55.00	88.63	181.63	226
	60min	45	0.38	1.68	0.21	0.03	59.56	108.44	231.78	177
	120min	50	0.21	1.8	0.24	0.02	130.60	111.60	236.80	107
	第一天	50	0.42	1.48	0.21	0.05	110.17	87.83	269.50	108
	第七天	50	0.14	1.08	0.01	-0.02	142.00	112.50	422.75	96
	第十四天	50	0.17	1.62	0.12	0.02	119.50	116.00	270.25	94

Table S9 ECG results after single oral administration of TA (No.: 71192F, 20#)

表 15　灯台叶碱提取物 Beagle 犬灌胃给药急性毒性试验心电检查结果（71192F(20#)）

时期		P 波时限(ms)	P 波振幅(mV)	R 波振幅(mV)	T 波振幅(mV)	ST 偏移量(mV)	P−R 间期(ms)	QRS 群间期(ms)	Q−T 间期(ms)	心率（次/分）
前期	第一次	40	0.25	1.26	0.16	-0.04	143.00	85.50	314.25	91
	第二次	50	0.24	1.70	0.17	0.01	122.67	140.33	290.33	97
	给药前	50	0.44	1.45	0.12	-0.04	89.83	121.17	284.17	111
给药后	30min	50	0.3	1.63	0.18	-0.02	78.40	137.80	291.60	127
	60min	50	1.08	1.49	0.1	0.01	108.67	105.33	226.33	148
	120min	50	0.28	1.86	0.05	-0.07	92.17	142.67	282.33	123
	第一天	45	0.25	1.71	0.11	-0.07	79.50	131.75	277.00	109
	第七天	50	0.26	1.08	0.06	-0.12	100.00	98.25	330.75	115
	第十四天	50	0.19	1.63	0.1	-0.07	90.00	137.00	288.00	70

Table S10 The urine and feces results after single oral administration of TA

表 16　灯台叶碱提取物 Beagle 犬灌胃给药急性毒性试验尿粪检查结果

项目	62244M(1#)					71192F(20#)				
	给药前		给药后			给药前		给药后		
	第 1 次	第 2 次	第 1 天	第 7 天	第 14 天	第 1 次	第 2 次	第 1 天	第 7 天	第 14 天
GLU	-	微量	微量	-	-	-	-	-	微量	-
BIL	-	1+	1+	-	1+	-	1+	-	2+	-
KET	-	-	-	-	-	-	-	-	-	-
SG	1.015	1.010	1.010	1.015	1.010	1.010	1.010	1.010	1.010	1.010
BLO	3+	微量-完整	-	1+	微量-完整	2+	-	1+	-	2+
PH	8.5	>=9.0	>=9.0	7.0	>=9.0	7.0	8.5	8.5	>=9.0	8.5
PRO	2+	3+	3+	微量	2+	微量	2+	1+	3+	2+
URO	16umol/L	16 umol/L	16 umol/L	3.2umol/L	3.2umol/L	3.2umol/L	16 umol/L	3.2umol/L	33 umol/L	3.2umol/L
NIT	-	+	+	+	-	+	+	+	+	+
WBC	3+	3+	1+	3+	3+	微量	微量	微量	1+	微量
粪潜血	-	-	-	-	-	-	-	-	-	-

注：-示阴性结果，±示假阳性，+示阳性。

Notes:

- negative, ± false positive, + positive

S4. The results of sub-chronic toxicity on beagle dogs

Table S11 Food intake after treatment with TA for 13 weeks via group comparison

表 6　Beagle 犬每周摄食量总评

	周次	空白对照组				低剂量组（20mg/kg.bw）				中剂量组（60mg/kg.bw）				高剂量组（120mg/kg.bw）			
		A	B	C	R 值	A	B	C	R 值	A	B	C	R 值	A	B	C	R 值
给药期 (n=6)	1	41	1	0	0.50	42	0	0	0.51	42	0	0	0.51	42	0	0	0.51
	2	40	2	0	0.50	39	3	0	0.49	42	0	0	0.52	42	0	0	0.52
	3	40	2	0	0.50	42	0	0	0.52	42	0	0	0.52	42	0	0	0.52
	4	42	0	0	0.50	42	0	0	0.50	42	0	0	0.50	42	0	0	0.50
	5	39	3	0	0.50	42	0	0	0.54	42	0	0	0.54	42	0	0	0.54
	6	42	0	0	0.50	42	0	0	0.50	42	0	0	0.50	42	0	0	0.50
	7	41	0	1	0.50	40	2	0	0.49	42	0	0	0.51	42	0	0	0.51
	8	41	1	0	0.50	41	1	0	0.50	42	0	0	0.51	42	0	0	0.51
	9	38	4	0	0.50	41	1	0	0.54	42	0	0	0.55	42	0	0	0.55
	10	40	2	0	0.50	42	0	0	0.52	42	0	0	0.52	42	0	0	0.52
	11	41	1	0	0.50	42	0	0	0.51	42	0	0	0.51	42	0	0	0.51
	12	40	2	0	0.50	42	0	0	0.52	42	0	0	0.52	42	0	0	0.52
	13	42	0	0	0.50	42	0	0	0.50	42	0	0	0.50	42	0	0	0.50
恢复期（n=2）	1	14	0	0	0.50	14	0	0	0.50	14	0	0	0.50	14	0	0	0.50
	2	14	0	0	0.50	14	0	0	0.50	14	0	0	0.50	14	0	0	0.50
	3	14	0	0	0.50	14	0	0	0.50	14	0	0	0.50	13	1	0	0.50
	4	14	0	0	0.50	14	0	0	0.50	14	0	0	0.50	14	0	0	0.50

多组等级资料分析：各组间比较无显著性差异（p>0.05）；

注：1. A、B、C 为摄食量评分等级：A—全部吃完或仅剩少量；B—剩余 1/3～1/2 量；C—未吃或仅吃少量；

2. R值为Ridit法平均分值；

Notes:

Multi-group grade data analysis, there was no significant difference between the groups ($p > 0.05$). R value was the average score of Ridit method.

A. The food was eaten up or left a small amount.

B. The rest food was 1/3-1/2.

C. The food was not eaten at all or only a little.

Table S12 Body weights after TA treatment for 13 weeks via group- and self- comparison

表 9　Beagle 犬给药前和给药期、恢复期各周平均体重变化（$\bar{x}$ ±SD，kg）

	周次	空白对照组	低剂量组（20mg/kg.bw）	中剂量组（60mg/kg.bw）	高剂量组（120mg/kg.bw）
给药前（n=6）	—	7.53±0.62	7.39±0.42	7.39±0.27	7.37±0.36
给药期（n=6）	0	7.68±0.72	7.68±0.64	7.66±0.11♥	7.71±0.37♥
	1	8.07±0.75♥	8.14±0.66♥♥	8.09±0.37♥♥	8.02±0.38♥♥
	2	8.35±0.80♥♥	8.38±0.83♥♥	8.31±0.29♥♥	8.24±0.49♥♥
	3	8.36±0.85♥♥	8.52±1.00♥	8.41±0.32♥♥	8.29±0.54♥♥
	4	8.65±0.92♥♥	8.79±1.04♥♥	8.70±0.32♥♥	8.50±0.57♥♥
	5	8.89±1.00♥♥	8.94±1.18♥♥	8.75±0.35♥♥	8.59±0.61♥♥
	6	8.97±1.12♥♥	8.90±1.20♥	8.84±0.35♥♥	8.59±0.64♥♥
	7	9.06±1.18♥	9.06±1.40♥	8.87±0.38♥♥	8.66±0.66♥♥
	8	9.30±1.25♥♥	9.25±1.46♥	9.05±0.42♥♥	8.72±0.77♥♥
	9	9.29±1.30♥♥	9.31±1.52♥	9.10±0.40♥♥	8.80±0.74♥♥
	10	9.26±1.38♥	9.44±1.54♥	9.15±0.45♥♥	8.84±0.78♥♥
	11	9.25±1.35♥	9.46±1.56♥	9.17±0.46♥♥	9.12±1.04♥
	12	9.17±1.30♥	9.56±1.60♥	9.16±0.54♥♥	8.92±0.80♥♥
	13	9.32±1.31♥♥	9.55±1.63♥	9.14±0.50♥♥	8.84±0.75♥♥
恢复期（n=2）	1	9.30±0.34	10.44±1.66	9.56±0.53	8.76±1.36
	2	9.26±0.28	10.26±1.65	9.56±0.62	8.79±1.51
	3	9.06±0.35	10.07±1.60	9.17±0.41	8.56±1.46
	4	9.28±0.37	10.21±1.50	9.43±0.50	8.69±1.40

方差分析：各组间比较无显著性差异（p>0.05）；

配对 t 检验：♥/♥♥与给药前自身对照比较有显著性差异（p <0.05/0.01）；

注：1. 给药前 $\bar{x}$ ±SD 为各组动物给药前第 1、2 周体重实测值平均值统计结果；

2. 恢复期观察每组动物数仅为两只，故不再做统计学比较，仅以 $\bar{x}$ ±SD 表示；

Notes:

Variance analysis, there was no significant difference between the groups ($p > 0.05$).

Pairwise T-test: ♥/♥♥ $p < 0.05/0.01$ self-comparison with before administration.

The mean ± SD represented the mean value of body weight at week -1 and -2 before administration.

During the recovery period, only two animals were observed in each group, so statistical comparison was no longer performed and was expressed as mean ± SD.

Table S13 Temperatures after TA treatment for 13 weeks via group- and self- comparison

表 11　Beagle 犬给药前后不同时期平均体温变化（$\bar{x}$ ±SD，℃）

组别	剂量（mg/kg.bw）	给药前	给药 5 周	给药 9 周	给药 13 周	停药恢复期 4 周
空白对照	—	38.87±0.21	38.67±0.29	38.72±0.31	39.07±0.44	39.15±0.35
低剂量组	20	38.98±0.25	38.58±0.32♥♥	38.63±0.20♥	38.65±0.48	38.50±0.14
中剂量组	60	38.78±0.13	38.52±0.39	38.67±0.24	38.83±0.48	38.40±0.14
高剂量组	120	38.77±0.36	38.55±0.43	38.60±0.52	38.77±0.47	38.95±0.49

方差分析：各组间比较无显著性差异（p>0.05）；

配对 t 检验：♥/♥♥与给药前自身对照比较有显著性差异（p<0.05/0.01）；

注：1. 给药前 $\bar{x}$ ±SD 为给药前第 1、2 周体温实测值平均值统计结果；

2. 恢复期观察每组动物数仅为两只，故不再做统计学比较，仅以 $\bar{x}$ ±SD 表示；

Notes:

Variance analysis, there was no significant difference between the groups ($p > 0.05$).

Pairwise T-test: ♥/♥♥ $p < 0.05/0.01$ self-comparison with before administration.

The mean ± SD represented the mean value of body weight at week -11 and -2 before administration.

During the recovery period, only two animals were observed in each group, so statistical comparison was no longer performed and was expressed as mean ± SD.

Table S14 Ophthalmological examination before treatment with TA

表 12　Beagle 犬给药前第 2 周眼底检查结果

组别	动物编号	左眼检测项目				右眼检测项目			
		视乳头	视网膜	血管	黄斑区	视乳头	视网膜	血管	黄斑区
空白对照组	62322F	圆、边界清	灰褐色	走形正常，A：V=1:2	反光弥散	圆、边界清	灰褐色	走形正常，A：V=1:2	反光弥散
	62717 F	圆、边界清	灰褐色	走形正常，A：V=1:3	反光弥散	圆、边界清	灰褐色	走形正常，A：V=1:3	反光弥散
	62570 F	圆、边界清	灰褐色	走形正常，A：V=1:3	反光弥散	圆、边界清	灰褐色	走形正常，A：V=1:3	反光弥散
	62455 M	圆、边界清	灰褐色	走形正常，A：V=1：3	反光弥散、色素多	圆、边界清	灰褐色	走形正常，A：V=1：3	反光弥散
	71036 M	圆、界清	灰红色	走形正常，A：V=1：3	反光弥散	圆、界清	灰红色	走形正常，A：V=1：3	反光弥散
	71074 M	圆、界清	灰红色	走形正常，A：V=1：3	反光弥散	圆、界清	灰红色	走形正常，A：V=1：3	反光弥散
低剂量组	62386 F	圆、小、界清	灰褐色	走形正常, A：V=1：3	反光弥散	圆、界清	灰褐色	走形正常, A：V=1：3	反光弥散
	62465 F	圆、大、界清	灰红色	走形正常，A：V=1：3	反光弥散	圆、大、界清	灰红色	走形正常，A：V=1：3	反光弥散
	62567 F	圆、界清	灰褐色	走形正常，A：V=1：3	反光弥散	圆、界清	灰褐色	走形正常，A：V=1：3	反光弥散
	62540 M	圆、界清	灰褐色	走形正常，A：V=1：3	反光弥散	圆、界清	灰褐色	走形正常，A：V=1：3	反光弥散
	62574 M	圆、界清	灰褐色	走形正常，A：V=1：3	反光弥散	圆、界清	灰褐色	走形正常，A：V=1：3	反光弥散
	71075 M	圆、界清	灰褐色	走形正常，A：V=1：3	反光弥散	圆、界清	灰褐色	走形正常，A：V=1：3	反光弥散

续表 12：

组别	动物编号	左眼检测项目				右眼检测项目			
		视乳头	视网膜	血管	黄斑区	视乳头	视网膜	血管	黄斑区
中剂量组	71013 F	圆、小、界清	灰褐色	走形正常，A：V=1：3	反光弥散	圆、界清	灰褐色	走形正常，A：V=1：3	反光弥散
	71088 F	圆、大、界清	灰红色	走形正常，A：V=1：2	反光弥散	圆、大、界清	灰红色	走形正常，A：V=1：2	反光弥散
	62490 F	圆、界清	红润	走形正常，A：V=1：2	反光弥散	圆、界清	红润	走形正常，A：V=1：2	反光弥散
	71195 M	圆、界清	灰褐色	走形正常，A：V=1：3	反光弥散	圆、界清	灰褐色	走形正常，A：V=1：3	反光弥散
	71007 M	圆、小、界清	灰褐色	走形正常，A：V=1：3	反光弥散	圆、界清	灰褐色	走形正常，A：V=1：3	反光弥散
	62541 M	圆、大、界清	灰红色	走形正常，A：V=1：2	反光弥散	圆、大、界清	灰红色	走形正常，A：V=1：2	反光弥散
高剂量组	62412 F	圆、界清	灰褐色	走形正常，A：V=1：3	反光弥散	圆、界清	灰褐色	走形正常，A：V=1：3	反光弥散
	62543 F	圆、界清	灰褐色	走形正常，A：V=1：3	反光弥散	圆、界清	灰红色	走形正常，A：V=1：3	反光弥散
	62610 F	圆、界清	灰红色	走形正常，A：V=1：2	反光弥散	圆、界清	灰红色	走形正常，A：V=1：2	反光弥散
	71112 M	圆、小、界清	灰褐色	走形正常，A：V=1：3	反光弥散	圆、界清	灰褐色	走形正常，A：V=1：3	反光弥散
	71066 M	圆、界清	灰褐色	走形正常，A：V=1：3	反光弥散	圆、大、界清	灰褐色	走形正常，A：V=1：3	反光弥散
	71262 M	圆、界清	灰褐色	走形正常，A：V=1：3	反光弥散	圆、界清	灰褐色	走形正常，A：V=1：3	反光弥散

Table S15 Ophthalmological examination after treatment with TA for 13 weeks

表 13　Beagle 犬给药第 13 周眼底检查结果

组别	动物编号	左眼检测项目				右眼检测项目			
		视乳头	视网膜	血管	黄斑区	视乳头	视网膜	血管	黄斑区
空白对照组	62322F	圆、小、界清	灰红色	走形正常，A：V=1：3	反光弥散	圆、小、界清	灰红色	走形正常，A：V=1：3	反光弥散
	62717 F	圆、界清	灰褐色	走形正常，A：V=1：3	反光弥散	圆、大、界清	灰褐色	走形正常，A：V=1：3	反光弥散
	62570 F	圆、界清	红润	走形正常，A：V=1：2	反光弥散	圆、红润、界清	红润	走形正常，A：V=1：2	反光弥散
	62455 M	圆、边界清	灰褐色	走形正常，A：V=1：3	反光弥散	圆、边界清	灰褐色	走形正常，A：V=1：3	反光弥散
	71036 M	圆、大、边界清	灰褐色	走形正常，A：V=1：3	反光弥散	圆、大、边界清	灰褐色	走形正常，A：V=1：3	反光弥散
	71074 M	圆、大、边界清	灰褐色	走形正常，A：V=1：3	反光弥散	圆、大、边界清	灰褐色	走形正常，A：V=1：3	反光弥散
低剂量组	62386 F	圆、界清	灰红色	走形正常，A：V=1：2	反光弥散	圆、界清	灰红色	走形正常，A：V=1：2	反光弥散
	62465 F	圆、边界清	灰褐色	走形正常，A：V=1：3	反光弥散	圆、边界清	灰褐色	走形正常，A：V=1：3	反光弥散
	62567 F	圆、界清	灰红色	走形正常，A：V=1：3	反光弥散	圆、界清	灰红色	走形正常，A：V=1：3	反光弥散
	62540 M	圆、边界清	灰褐色	走形正常，A：V=1：3	反光弥散	圆、边界清	灰褐色	走形正常，A：V=1：3	反光弥散
	62574 M	圆、边界清	灰褐色	走形正常，A：V=1：3	反光正常	圆、边界清	灰褐色	走形正常，A：V=1：3	反光弥散
	71075 M	圆、边界清	灰褐色	走形正常，A：V=1：3	反光弥散	圆、边界清	灰褐色	走形正常，A：V=1：3	反光弥散

续表 13：

组别	动物编号	左眼检测项目				右眼检测项目			
		视乳头	视网膜	血管	黄斑区	视乳头	视网膜	血管	黄斑区
中剂量组	71013 F	圆、边界清	灰红色	走形正常，A：V=1：3	反光弥散	圆、边界清	灰红色	走形正常，A：V=1：3	反光弥散
	71088 F	圆、边界清	灰红色	走形正常，A：V=1：3	反光弥散	圆、边界清	灰红色	走形正常，A：V=1：3	反光弥散
	62490 F	圆、红润、边界清	红润	走形正常，A：V=1：2	反光弥散	圆、红润、边界清	红润	走形正常，A：V=1：2	反光弥散
	71195 M	圆、边界清	灰褐色	走形正常，A：V=1：2	反光弥散	圆、边界清	灰褐色	走形正常，A：V=1：2	反光弥散
	71007 M	圆、大、边界清	灰褐色	走形正常，A：V=1：3	反光弥散	圆、大、边界清	灰褐色	走形正常，A：V=1：3	反光弥散
	62541 M	圆、边界清	灰褐色	走形正常，A：V=1：3	反光弥散	圆、边界清	灰褐色	走形正常，A：V=1：3	反光弥散
高剂量组	62412 F	圆、边界清	灰红色	走形正常，A：V=1：3	反光弥散	圆、边界清	灰红色	走形正常，A：V=1：3	反光弥散
	62543 F	圆、边界清	灰褐色	走形正常，静脉迂曲 A：V=1：3	反光弥散	圆、边界清	灰褐色	走形正常，静脉迂曲 A：V=1：3	反光弥散
	62610 F	圆、边界清	灰褐色	走形正常，A：V=1：3	反光弥散	圆、边界清	灰褐色	走形正常，A：V=1：3	反光弥散
	71112 M	圆、边界清	灰红色	走形正常，A：V=1：2	反光弥散	圆、边界清	灰红色	走形正常，A：V=1：2	反光弥散
	71066 M	圆、边界清	灰红色	走形正常，A：V=1：2	反光弥散	圆、边界清	灰红色	走形正常，A：V=1：2	反光弥散
	71262 M	圆、边界清	灰褐色	走形正常，A：V=1：3	反光弥散	界清，红润	灰褐色	走形正常，A：V=1：3	反光弥散

Table S16 Ophthalmological examination after withdrawal TA for 4 weeks

表 14　Beagle 犬停药恢复 4 周眼底检查结果

组别	动物编号	左 眼 检 测 项 目				右 眼 检 测 项 目			
		视乳头	视网膜	血管	黄斑区	视乳头	视网膜	血管	黄斑区
空白对照组	62570 F	圆、边界清	灰褐色	走形正常，A：V=1：3	反光弥散	圆、边界清	灰褐色	走形正常，A：V=1：3	反光弥散
	62455 M	圆、边界清	灰红色	走形正常，A：V=1：3	反光弥散	圆、边界清	灰红色	走形正常，A：V=1：3	反光弥散
低剂量组	62386 F	圆、大、边界清	灰红色	走形正常，A：V=1：3	反光弥散	圆、大、边界清	灰红色	走形正常，A：V=1：3	反光弥散
	71075 M	圆、边界清	灰红色	走形正常，A：V=1：3	反光弥散	圆、边界清	灰红色	走形正常，A：V=1：3	反光弥散
中剂量组	62490 F	圆、小、边界清	红润	走形正常，A：V=1：2	反光弥散	圆、小、边界清	红润	走形正常，A：V=1：2	反光弥散
	62541 M	圆、大、边界清	灰红色	走形正常，A：V=1：2	反光弥散	圆、大、边界清	灰红色	走形正常，A：V=1：2	反光弥散
高剂量组	62412 F	圆、边界清	灰红色	走形正常，A：V=1：2	反光弥散	圆、边界清	灰红色	走形正常，A：V=1：2	反光弥散
	71262 M	圆、边界清	灰褐色	走形正常，A：V=1：2	反光弥散	圆、边界清	灰褐色	走形正常，A：V=1：2	反光弥散

Table S17 Urinalysis before the administration of TA for -1 week

表 15　　Beagle 犬给药前第 1 周尿液检查结果

组别	动物编号	尿液检查项目								
		GLU	BIL	KET	SG	BLO	pH	PRO	URO	WBC
空白对照组	62322F	−	−	−	1.010	+++	7.5	−	3.2	<+
	62717 F	−	−	−	1.015	++	7.5	+	3.2	<+
	62570 F	−	−	−	1.010	+++	7.0	+	3.2	−
	62455 M	−	−	−	1.015	<+	8.5	+	3.2	+++
	71036 M	−	−	−	1.025	+	7.0	++	3.2	<+
	71074 M	−	−	−	1.010	+++	7.5	++	3.2	+++
低剂量组	62386 F	−	−	−	1.015	+++	8.0	−	3.2	−
	62465 F	−	−	−	1.015	+++	8.0	+	3.2	<+
	62567 F	−	−	−	1.010	+	8.5	−	3.2	+
	62540 M	−	−	−	1.020	+	6.0	−	3.2	<+
	62574 M	−	−	−	1.030	−	7.0	+	3.2	<+
	71075 M	−	−	−	1.020	+	6.5	<+	3.2	−
中剂量组	71013 F	−	−	−	1.010	<+	9.0	++	3.2	<+
	71088 F	−	−	−	1.010	<+	9.0	++	3.2	−
	62490 F	−	−	−	1.015	++	8.5	++	3.2	+
	71195 M	−	−	−	1.015	<+	8.5	++	3.2	−
	71007 M	−	−	−	1.020	<+	7.0	−	3.2	−
	62541 M	−	−	−	1.015	++	7.5	+	3.2	+++
高剂量组	62412 F	−	−	−	1.010	+++	7.5	−	3.2	−
	62543 F	−	−	−	1.020	+++	7.0	+	3.2	<+
	62610 F	−	−	−	1.015	+++	8.0	+	16	−
	71112 M	−	−	−	1.015	<+	7.0	<+	3.2	+
	71066 M	−	−	−	1.030	+	7.0	+	3.2	<+
	71262 M	−	−	−	1.010	+	8.5	++	3.2	+

注：1. 给药前第 1 周即前期 1；

2. 定量指标：尿比重（SG），pH 值、尿胆原（URO）；

3. 半定量指标：尿糖(GLU) Negative 为：(—)，5.5mmol/L 为：微量(<+)，14.0mmol/L 为：(+)，28.0mmol/L 为：(++)，≥55.0mmol/L 为：(+++)；

尿蛋白（PRO）Negative 为：(—)，0.15g/L 为：微量(<+)，0.3g/L 为：(+)，1.0g/L 为：++，≥3.0g/L 为：(+++)；

尿酮体（KET）Negative 为：(—)，Trace 为：微量(<+)，1.5mmol/L 为：(+)，3.9mmol/L 为：(++)，7.8mmol/L 为：(+++)，≥15.6mmol/L 为：(++++)；

尿潜血（BLO）Negative 为：(—)，Trace-lysed 或 Trace-intact 为：微量(<+)，Ca25Ery/ul 为：(+)，Ca80Ery/ul 为：(++)，Ca200Ery/ul 为：(+++)；

白细胞（WBC）Negative 为：(—)，Ca15Leu/ul 为：微量(<+)，Ca70Leu/ul 为：(+)，Ca125Leu/ul 为：(++)，Ca500Leu/ul 为：为：(+++)；

4. 定性指标：尿外观、尿胆红素（BIL）Negative 为：(—)，Small 为：(+)，Moderate 为：(++)，arge 为：(+++)；

Notes:

1. The urinalysis represented the quarantine period (-1 week);

2. Quantitative indices：Specific gravity (SG),   pH value, Urobilinogen (URO);

3. Semiquantitative indices:

Glucose (GLU), —: Negative: <＋: 5.5 mmol/L, +: 14.0 mmol/L, ++: 28.0 mmol/L, +++: ≥ 55.0 mmol/L

Protein (PRO), —: Negative, <＋: 0.15 g/L, +: 0.3 g/L, ++: 1.0 g/L, +++: ≥3.0 g/L

Ketone body (KET), —: Negative, <＋: Trace, +: 1.5 mmol/L, ++: 3.9 mmol/L, +++: 7.8 mmol/L, ++++: ≥15.6 mmol/L

Occult blood (BLO), —: Negative, <＋: Trace-lysed or Trace-intact, +: Ca25Ery/uL, ++: Ca80Ery/uL, +++: Ca200Ery/uL

White blood cell (WBC), —: Negative, <＋, Ca15Leu/uL, +: Ca70Leu/uL, ++: Ca125Leu/uL, +++: Ca500Leu/uL

4. Qualitative indices: Bilirubin (BIL), —: Negative, +: Small, ++, Moderate, +++: large

Table S18 Urinalysis before the administration of TA for -2 week

表 16　Beagle 犬给药前第 2 周尿液检查结果

组别	动物编号	尿液检查项目								
		GLU	BIL	KET	SG	BLO	pH	PRO	URO	WBC
空白对照组	62322F	－	－	－	1.010	<+	8.5	+++	3.2	+
	62717 F	－	－	－	1.025	－	6.5	+	3.2	－
	62570 F	－	－	－	1.030	++	6.0	++	3.2	+++
	62455 M	－	－	－	1.010	+	9.0	+	3.2	+++
	71036 M	－	－	－	1.020	－	7.0	+	3.2	+
	71074 M	－	－	－	1.010	+++	8.5	－	3.2	+++
低剂量组	62386 F	－	－	－	1.015	+++	7.0	+	3.2	<+
	62465 F	－	+	－	1.025	++	7.0	+	3.2	<+
	62567 F	－	－	－	1.015	++	8.5	<+	3.2	－
	62540 M	<+	－	－	1.010	+	9.0	+++	16	<+
	62574 M	－	－	－	1.020	++	7.0	++	3.2	+++
	71075 M	－	－	－	1.020	+	7.0	++	3.2	<+
中剂量组	71013 F	－	－	－	1.010	－	9.0	++	3.2	<+
	71088 F	－	－	－	1.010	<+	8.5	+++	3.2	－
	62490 F	－	－	－	1.015	++	8.5	+	3.2	－
	71195 M	－	－	－	1.025	<+	6.5	+	3.2	+++
	71007 M	－	－	－	1.010	－	8.5	++	3.2	+++
	62541 M	－	－	－	1.020	<+	7.0	<+	3.2	<+
高剂量组	62412 F	－	－	－	1.010	+++	8.5	+	3.2	<+
	62543 F	<+	+	<+	1.025	++	7.0	－	3.2	+++
	62610 F	－	－	－	1.010	+	8.5	<+	3.2	<+
	71112 M	－	－	－	1.010	+	8.5	++	3.2	+++
	71066 M	－	－	－	1.015	+	7.5	++	3.2	+++
	71262 M	－	－	－	1.010	+	7.5	+	3.2	+++

Notes:

1. The urinalysis represented the quarantine period (-1 week);

2. Quantitative indices：Specific gravity (SG),　pH value, Urobilinogen (URO);

3. Semiquantitative indices:

Glucose (GLU), —: Negative:　<＋: 5.5 mmol/L, +: 14.0 mmol/L, ++: 28.0 mmol/L, +++: ≥ 55.0 mmol/L

Protein (PRO), —: Negative, <＋: 0.15 g/L, +: 0.3 g/L, ++: 1.0 g/L, +++: ≥3.0 g/L

Ketone body (KET), —: Negative, <＋: Trace, +: 1.5 mmol/L, ++: 3.9 mmol/L, +++: 7.8 mmol/L, ++++: ≥15.6 mmol/L

Occult blood (BLO), —: Negative, <＋: Trace-lysed or Trace-intact, +: Ca25Ery/uL, ++: Ca80Ery/uL, +++: Ca200Ery/uL

White blood cell (WBC), —: Negative, <+, Ca15Leu/uL, +: Ca70Leu/uL, ++: Ca125Leu/uL, +++: Ca500Leu/uL

4. Qualitative indices: Bilirubin (BIL), —: Negative, +: Small, ++, Moderate, +++: large

Table S19 Urinalysis after the administration of TA for 5 weeks

表 17　Beagle 犬给药第 5 周尿液检查结果

组别	动物编号	尿液检查项目								
		GLU	BIL	KET	SG	BLO	pH	PRO	URO	WBC
空白对照组	62322F	−	−	−	1.010	−	9.0	++	3.2	<+
	62717 F	−	−	−	1.025	<+	7.0	+	3.2	+
	62570 F	−	−	−	1.010	<+	8.5	+++	16	+++
	62455 M	−	−	−	1.010	−	9.0	++	3.2	+++
	71036 M	−	+	−	1.015	<+	8.5	+++	16	+++
	71074 M	−	−	−	1.015	+++	8.5	+	3.2	+
低剂量组	62386 F	−	−	<+	1.010	−	8.5	+++	16	+
	62465 F	−	+	−	1.010	−	9.0	+++	16	−
	62567 F	−	−	−	1.015	−	7.5	+	3.2	−
	62540 M	−	−	−	1.020	++	7.0	++	3.2	<+
	62574 M	−	−	<+	1.010	<+	8.5	+++	16	+++
	71075 M	−	+	−	1.010	−	8.5	+++	16	+++
中剂量组	71013 F	−	−	−	1.010	−	9.0	++	3.2	<+
	71088 F	−	−	−	1.010	−	8.5	+++	16	−
	62490 F	−	−	−	1.010	−	9.0	++	3.2	<+
	71195 M	−	−	−	1.010	+	8.5	++	3.2	+++
	71007 M	−	−	−	1.010	−	9.0	+++	16	+
	62541 M	−	−	<+	1.015	+	8.0	++	3.2	+++
高剂量组	62412 F	−	−	−	1.020	++	7.0	+	3.2	−
	62543 F	−	−	−	1.010	+++	8.5	++	3.2	<+
	62610 F	−	−	−	1.010	++	7.0	<+	3.2	<+
	71112 M	−	−	−	1.010	+	8.5	+++	3.2	+++
	71066 M	−	−	−	1.015	+	7.0	<+	3.2	<+
	71262 M	−	−	−	1.010	−	8.5	++	3.2	+++

Notes:

1. The urinalysis represented the quarantine period (-1 week);
2. Quantitative indices：Specific gravity (SG),　pH value, Urobilinogen (URO);
3. Semiquantitative indices:

Glucose (GLU), —: Negative:　<＋: 5.5 mmol/L, +: 14.0 mmol/L, ++: 28.0 mmol/L, +++: ≥ 55.0 mmol/L

Protein (PRO), —: Negative, <＋: 0.15 g/L, +: 0.3 g/L, ++: 1.0 g/L, +++: ≥3.0 g/L

Ketone body (KET), —: Negative, <＋: Trace, +: 1.5 mmol/L, ++: 3.9 mmol/L, +++: 7.8 mmol/L, ++++: ≥15.6 mmol/L

Occult blood (BLO), —: Negative, <＋: Trace-lysed or Trace-intact, +: Ca25Ery/uL, ++: Ca80Ery/uL, +++: Ca200Ery/uL

White blood cell (WBC), —: Negative, <+, Ca15Leu/uL, +: Ca70Leu/uL, ++: Ca125Leu/uL, +++: Ca500Leu/uL

4. Qualitative indices: Bilirubin (BIL), —: Negative, +: Small, ++, Moderate, +++: large

Table S20 Urinalysis after the administration of TA for 9 weeks

表 18　Beagle 犬给药第 9 周尿液检查结果

组别	动物编号	尿液检查项目								
		GLU	BIL	KET	SG	BLO	pH	PRO	URO	WBC
	62322F	—	—	—	1.015	+++	7.0	++	3.2	—
	62717 F	—	—	—	1.010	++	7.5	+	3.2	<+
空白对照组	62570 F	<+	+	—	1.010	—	8.5	+++	16	<+
	62455 M	—	—	—	1.010	—	8.5	+++	3.2	<+
	71036 M	—	—	—	1.020	+++	7.5	++	3.2	+++
	71074 M	—	—	—	1.015	+++	9.0	++	3.2	+
	62386 F	—	—	—	1.015	+	7.0	+	3.2	<+
	62465 F	<+	+	<+	1.010	++	8.5	+++	16	—
低剂量组	62567 F	—	—	—	1.010	+++	8.5	++	3.2	—
	62540 M	—	—	—	1.015	—	8.5	++	3.2	+
	62574 M	—	—	—	1.025	<+	7.0	+	3.2	+
	71075 M	—	—	<+	1.010	—	9.0	+++	16	<+
	71013 F	—	—	<+	1.015	<+	8.5	++	16	+++
	71088 F	—	+	+	1.015	<+	8.5	+++	3.2	<+
	62490 F	—	—	—	1.010	++	7.0	++	3.2	—
中剂量组	71195 M	—	—	—	1.010	++	7.5	+	3.2	+
	71007 M	—	—	—	1.025	+	7.5	+++	16	<+
	62541 M	—	—	<+	1.015	—	7.5	++	16	—
	62412 F	—	—	—	1.010	+++	8.5	++	3.2	—
	62543 F	—	—	<+	1.025	—	7.0	+	3.2	—
	62610 F	—	+	—	1.015	+++	7.5	++	3.2	<+
高剂量组	71112 M	—	—	—	1.025	+	7.0	++	3.2	+++
	71066 M	—	—	—	1.015	+	8.5	<+	3.2	<+
	71262 M	—	—	—	1.010	—	8.5	+++	16	+++

[illegible]

Notes:

1. The urinalysis represented the quarantine period (-1 week);
2. Quantitative indices：Specific gravity (SG),　pH value, Urobilinogen (URO);
3. Semiquantitative indices:

Glucose (GLU), —: Negative:　<＋: 5.5 mmol/L, +: 14.0 mmol/L, ++: 28.0 mmol/L, +++: ≥ 55.0 mmol/L

Protein (PRO), —: Negative, <＋: 0.15 g/L, +: 0.3 g/L, ++: 1.0 g/L, +++: ≥3.0 g/L

Ketone body (KET), —: Negative, <＋: Trace, +: 1.5 mmol/L, ++: 3.9 mmol/L, +++: 7.8 mmol/L, ++++: ≥15.6 mmol/L

Occult blood (BLO), —: Negative, <＋: Trace-lysed or Trace-intact, +: Ca25Ery/uL, ++: Ca80Ery/uL, +++: Ca200Ery/uL

White blood cell (WBC), —: Negative, <+, Ca15Leu/uL, +: Ca70Leu/uL, ++: Ca125Leu/uL, +++: Ca500Leu/uL

4. Qualitative indices: Bilirubin (BIL), —: Negative, +: Small, ++, Moderate, +++: large

Table S21 Urinalysis after the administration of TA for 13 weeks

表 19　Beagle 犬给药第 13 周尿液检查结果

组别	动物编号	尿液检查项目								
		GLU	BIL	KET	SG	BLO	pH	PRO	URO	WBC
空白对照组	62322F	−	+	−	1.010	−	9.0	++	16	−
	62717 F	−	+	+	1.020	+++	8.5	++	3.2	+++
	62570 F	−	−	−	1.015	+	7.5	+	3.2	+
	62455 M	−	+	−	1.010	<+	8.5	++	3.2	+++
	71036 M	−	−	−	1.015	+++	7.0	+	3.2	<+
	71074 M	−	−	−	1.010	+++	8.5	++	3.2	+++
低剂量组	62386 F	−	+	−	1.025	−	6.0	<+	3.2	<+
	62465 F	−	−	−	1.010	++	7.5	−	3.2	−
	62567 F	−	−	−	1.010	−	9.0	++	3.2	−
	62540 M	−	+	<+	1.010	+	6.0	++	3.2	<+
	62574 M	−	−	−	1.010	−	8.5	+++	16	+++
	71075 M	−	−	−	1.010	−	9.0	++	16	+++
中剂量组	71013 F	−	−	<+	1.010	−	8.5	+++	16	+
	71088 F	−	+	+	1.010	−	8.5	++	16	+
	62490 F	−	−	−	1.005	+++	8.0	++	3.2	<+
	71195 M	−	+	−	1.015	−	7.5	+	3.2	<+
	71007 M	−	−	−	1.010	<+	8.5	+++	16	<+
	62541 M	−	+	−	1.010	+	8.5	++	3.2	+++
高剂量组	62412 F	−	+	−	1.010	−	8.5	++	3.2	<+
	62543 F	−	+	−	1.030	++	6.0	+	3.2	<+
	62610 F	−	−	−	1.005	+++	8.5	−	3.2	<+
	71112 M	−	+	−	1.010	−	9.0	++	3.2	+++
	71066 M	−	+	<+	1.025	−	7.0	++	3.2	<+
	71262 M	−	+	−	1.005	−	9.0	+++	16	+

[illegible]

Notes:

1. The urinalysis represented the quarantine period (-1 week);
2. Quantitative indices：Specific gravity (SG),　pH value, Urobilinogen (URO);
3. Semiquantitative indices:

Glucose (GLU), —: Negative:　<＋: 5.5 mmol/L, +: 14.0 mmol/L, ++: 28.0 mmol/L, +++: ≥ 55.0 mmol/L

Protein (PRO), —: Negative, <＋: 0.15 g/L, +: 0.3 g/L, ++: 1.0 g/L, +++: ≥3.0 g/L

Ketone body (KET), —: Negative, <＋: Trace, +: 1.5 mmol/L, ++: 3.9 mmol/L, +++: 7.8 mmol/L, ++++: ≥15.6 mmol/L

Occult blood (BLO), —: Negative, <＋: Trace-lysed or Trace-intact, +: Ca25Ery/uL, ++: Ca80Ery/uL, +++: Ca200Ery/uL

White blood cell (WBC), —: Negative, <+, Ca15Leu/uL, +: Ca70Leu/uL, ++: Ca125Leu/uL, +++: Ca500Leu/uL

4. Qualitative indices: Bilirubin (BIL), —: Negative, +: Small, ++, Moderate, +++: largeD

Table S22 Urinalysis after the withdrawal of TA for 4 weeks

表 20　Beagle 犬停药恢复 4 周尿液检查结果

组别	动物编号	尿液检查项目								
		GLU	BIL	KET	SG	BLO	pH	PRO	URO	WBC
空白对照组	62570 F	−	+	−	1.005	<+	9.0	++	3.2	<+
	62455 M	−	+	−	1.010	−	9.0	++	3.2	+
低剂量组	62386 F	−	+	−	1.010	−	8.5	+++	16	<+
	71075 M	−	−	<+	1.010	−	9.0	++	3.2	+++
中剂量组	62490 F	−	−	−	1.010	−	9.0	+++	3.2	<+
	62541 M	−	+	<+	1.015	<+	9.0	+++	16	+++
高剂量组	62412 F	−	−	<+	1.010	−	9.0	+++	16	<+
	71262 M	−	+	−	1.010	−	9.0	+++	16	+++

[illegible]

Notes:

1. The urinalysis represented the quarantine period (-1 week);

2. Quantitative indices：Specific gravity (SG),　pH value, Urobilinogen (URO);

3. Semiquantitative indices:

Glucose (GLU), —: Negative:　<＋: 5.5 mmol/L, +: 14.0 mmol/L, ++: 28.0 mmol/L, +++: ≥ 55.0 mmol/L

Protein (PRO), —: Negative, <＋: 0.15 g/L, +: 0.3 g/L, ++: 1.0 g/L, +++: ≥3.0 g/L

Ketone body (KET), —: Negative, <＋: Trace, +: 1.5 mmol/L, ++: 3.9 mmol/L, +++: 7.8 mmol/L, ++++: ≥15.6 mmol/L

Occult blood (BLO), —: Negative, <＋: Trace-lysed or Trace-intact, +: Ca25Ery/uL, ++: Ca80Ery/uL, +++: Ca200Ery/uL

White blood cell (WBC), —: Negative, <＋, Ca15Leu/uL, +: Ca70Leu/uL, ++: Ca125Leu/uL, +++: Ca500Leu/uL

4. Qualitative indices: Bilirubin (BIL), —: Negative, +: Small, ++, Moderate, +++: large

Table S23 SG, PH, and URO results during the study period via group- and self-comparison

表 21　Beagle 犬给药前后不同时期尿液 SG、PH、URO 组间和自身对照比较结果（$\bar{x}$ ±SD）

指标	组别	剂量（mg/kg.bw）	给药前	给药 5 周	给药 9 周	给药 13 周	停药恢复 4 周
SG	空白对照	—	1.02±0.004	1.01±0.006	1.01±0.004	1.01±0.004	1.01±0.004
	低剂量组	20	1.02±0.003	1.01±0.004♥	1.01±0.006	1.01±0.006	1.01±0.00
	中剂量组	60	1.02±0.006	1.01±0.002♥	1.02±0.005	1.01±0.009	1.01±0.004
	高剂量组	120	0.93±0.21	1.01±0.004	1.02±0.007	1.01±0.011	1.01±0.00
PH	空白对照	—	7.96±0.62	8.42±0.74	8.00±0.77	8.17±0.75	9.00±0.00
	低剂量组	20	7.50±0.77	8.17±0.75	8.08±0.86	7.67±1.40	8.75±0.35
	中剂量组	60	7.00±0.42	8.67±0.41♥♥	7.92±0.66♥	8.25±0.42♥♥	9.00±0.00
	高剂量组	120	6.92±1.59	7.75±0.82	7.83±0.75	8.00±1.22	9.00±0.00
URO（g/L）	空白对照	—	3.20±0.00	7.47±6.61	5.33±5.22	5.33±5.22	3.20±0.00
	低剂量组	20	5.33±3.30	11.73±6.61	7.47±6.61	7.47±6.61	9.60±9.05
	中剂量组	60	3.20±0.00	7.47±6.61	9.60±7.01	9.60±7.01	9.60±9.05
	高剂量组	120	2.93±0.65	3.20±0.00	5.33±5.22	5.33±5.22	16.00±0.00♥

方差分析：各组间比较无显著性差异（p>0.05）；

配对 t 检验：♥/♥♥与给药前自身对照比较有显著性差异（p <0.05/0.01）；

注：1. 给药前 $\bar{x}$ ±SD 为给药前第 1、2 周检查结果平均值统计结果；

2. 恢复期观察每组动物数仅为两只，故不再做统计学比较，仅以 $\bar{x}$ ±SD 表示；

Notes:

Variance analysis, there was no significant difference between the groups ($p > 0.05$).

Pairwise T-test: ♥/♥♥ $p < 0.05/0.01$ self-comparison with before administration.

The mean ± SD represented the mean value of urinalysis at week 1 and 2 before administration.

During the recovery period, only two animals were observed in each group, so statistical comparison was no longer performed and was expressed as mean ± SD.

Table S24 Feces examination during the study period via group- and self-comparison

表 22 Beagle 犬给药前后不同时期粪便检查结果

组别	动物编号	给药前		给药 13 周		停药恢复 4 周	
		潜血（OB）	寄生虫	潜血（OB）	寄生虫	潜血（OB）	寄生虫
空白对照组	62322 F	—	—	—	—		
	62717 F	+	—	—	—		
	62570 F	—	—	—	—	—	—
	62455 M	—	—	—	—	—	—
	71036 M	—	—	—	—		
	71074 M	+	—	—	—		
低剂量组	62386 F	+	—	—	—	—	—
	62465 F	—	—	—	—		
	62567 F	—	—	—	—		
	62540 M	—	—	—	—		
	62574 M	—	—	—	—		
	71075 M	+	—	—	—	—	—
中剂量组	71013 F	—	—	—	—		
	71088 F	—	—	—	—		
	62490 F	—	—	—	—	—	—
	71195 M	+	—	—	—		
	71007 M	—	—	—	—		
	62541 M	+	—	—	—	—	—
高剂量组	62412 F	+	—	—	—	—	—
	62543 F	++	—	—	—		
	62610 F	—	—	—	—		
	71112 M	—	—	—	—		
	71066 M	—	—	—	—		
	71262 M	—	—	—	—	—	—

注： OB：阴性(—)、弱阳性(±)、偶见、偶见阳性(+)、强阳性（++）；

寄虫卵：未见(—)、找到(±)、多(+)、较多(++)；

Notes:

Occult blood (OB), —: Negative, ±: weakly positive, +: occasionally positive, ++: strong positive

Parasite eggs, —: unobserved, ±: observed, +: many, ++: much more

Table S25 Electrocardiographic examination during the study period via group comparison

表 9　灯台叶碱提取物 3 个月长期毒性试验 Beagle 犬心电检测数据统计结果（$\bar{x}$±SD）

测定时间	组别	P 波时限 (ms)	P 波电压 (mv)	P-R 间期 (ms)	QRS 间期 (ms)	Q-T 间期 (ms)	S-T 段上抬 (mv)	R 波电压 (mv)	T 波 (mv)	心率 次/分（HR）
给药前 2 次 (n=12)	空白对照组	65.00±9.70	0.28±0.14	91.38±15.48	80.91±24.42	239.87±35.62	0.04±0.03	1.44±0.21	0.24±0.10	134.17±18.83
	低剂量组	67.75±10.88	0.23±0.04	82.44±12.12	72.41±19.21	209.14±24.29	0.04±0.02	1.43±0.23	0.36±0.18	148.50±19.95
	中剂量组	66.00±13.00	0.30±0.20	81.55±13.22	70.96±27.02	227.76±24.73	0.04±0.02	1.32±0.47	0.34±0.18	143.25±18.25
	高剂量组	66.58±8.27	0.27±0.16	84.03±19.05	84.51±20.99	226.64±25.13	0.03±0.03	1.24±0.25	0.22±0.12	137.00±19.72
给药后 第 5 周 (n=6)	空白对照组	61.00±8.74	0.26±0.08	83.81±11.68*	77.62±26.48	227.32±24.91	0.03±0.03	1.30±0.30	0.30±0.19	145.67±15.94
	低剂量组	72.00±14.14*	0.33±0.08	87.46±24.90	81.63±28.94	223.73±17.44	0.03±0.03	1.45±0.33	0.28±0.18	142.83±31.08
	中剂量组	71.67±12.80	0.28±0.08	94.94±21.50	70.85±25.09	249.42±40.24	0.03±0.03	1.28±0.40	0.27±0.18	136.33±26.08
	高剂量组	74.33±16.32	0.31±0.08	83.98±8.86*	93.90±28.80	285.60±54.80	0.05±0.06	1.20±0.21	0.20±0.18	135.50±12.88
给药后 第 9 周 (n=6)	空白对照组	57.00±8.92	0.40±0.41	81.62±18.42*	83.59±20.70	263.05±89.08	0.02±0.02	1.10±0.25	018±0.07	141.00±15.89
	低剂量组	52.00±7.16**	0.20±0.03	91.51±20.94	81.70±11.00	246.07±78.66	0.04±0.03	1.08±0.26	0.21±0.10	137.67±24.95
	中剂量组	67.67±8.80	0.24±0.05	92.28±14.30	75.75±12.66	211.44±20.01	0.04±0.03	0.89±0.51	0.36±0.17	145.50±24.42
	高剂量组	56.00±5.06	0.23±0.04	84.21±27.10	82.39±4.31	255.23±23.42	0.06±0.05	0.83±0.24	0.14±0.10	140.67±24.47
给药后 第 13 周 (n=6)	空白对照组	65.00±10.49	0.37±0.14	97.25±17.74	99.05±22.57	235.02±44.82	0.04±0.04	1.61±0.25	0.16±0.08	140.83±23.08
	低剂量组	56.67±10.33 Δ /**	0.36±0.29	95.30±20.53	99.96±23.21	222.00±34.46	0.04±0.04	1.56±0.27	0.20±0.12	152.50±27.18
	中剂量组	61.67±7.52	0.42±0.33	105.74±19.33	70.21±20.41	252.12±9.11	0.04±0.04	1.33±0.54	0.29±0.18	136.17±12.42
	高剂量组	70.00±7.07	0.27±0.10	92.51±9.58*	96.42±35.71	278.05±36.16	0.09±0.07	1.33±0.31	0.10±0.07	150.40±20.86
恢复期 (n=6)	空白对照组	60.00±14.14	0.30±0.06	94.93±2.22	103.62±22.46	265.36±4.76	0.01±0.01	1.55±0.07	0.08±0.02	136.00±1.41
	低剂量组	55.00±7.07*	0.24±0.05	86.26±14.04	72.86±15.60	211.02±19.31	0.01±0.00	1.22±0.02	0.32±0.09	155.00±5.66
	中剂量组	65.00±7.07	0.21±0.06	89.96±26.11	79.02±27.09	273.16±8.46	0.01±0.00	1.12±0.42	0.14±0.07	140.00±24.04
	高剂量组	55.00±7.07	0.27±0.11	82.16±3.37	110.16±29.61	297.71±31.01	0.04±0.07	1.36±0.14	0.12±0.08	143.00±14.14'

方差分析：*/**自身对照比较有显著性差异（p<0.05/0.01）；Δ/ΔΔ与同期空白组比较有显著性差异（p<0.05/0.01）。

注：给药前数据为采集 2 次基础值之平均值

Notes:

Variance analysis, there was no significant difference between the groups ($p > 0.05$).

Pairwise T-test: ♥/♥♥ $p < 0.05/0.01$ self-comparison with before administration.

The mean ± SD represented the mean value of urinalysis at week -1 and -2 before administration.

During the recovery period, only two animals were observed in each group, so statistical comparison was no longer performed and was expressed as mean ± SD.

Table S26 The results of bone marrow examination at different times via group comparison

表 49　Beagle 犬给药 13 周和停药恢复 4 周骨髓象各检查值组间比较结果（$\bar{x}$ ±SD，%）

检查时间	$\bar{x}$ ± SD / 组别	粒系											红系				其它			
		原始粒	早幼粒	中性粒	晚幼粒	杆状粒	分叶粒	嗜酸中幼粒	嗜酸晚幼粒	嗜酸杆状粒	嗜酸分叶粒	合计	早幼红	中幼红	晚幼红	合计	粒红比	淋巴细胞	单核细胞	浆细胞
给药 13 周	对照组	1.25	3.12	3.88	7.00	26.50	4.00	1.25	1.00	0.88	0.50	49.38	1.50	8.50	26.62	36.62	1.38	9.00	3.25	0.88
		0.29	1.11	0.48	2.04	3.24	1.08	0.29	0.00	0.25	0.00	4.99	0.41	2.48	4.61	5.17	0.32	0.82	0.96	1.11
	低剂量	1.12	2.25	5.50	6. 00	29. 88	5. 25	0. 75	1. 00	0. 50	0. 38	52. 62	1. 00	6. 38	24. 62	32. 00	2. 23	10. 25	3. 62	1. 00
		0.75	0.64	1.68	0. 91	8. 82	1. 44	0. 64	0. 41	0. 41	0. 48	12. 31	0. 71	2. 50	9.84	12. 80	1. 98	0. 64	0. 95	0. 71
	中剂量	1.00	2.00	4.25	6.50	28.25	4.38	0.88	0.88	0. 62	0.62	49.38	1.38	8.12	29.25	38.75	1.30	9.50	2.88	1.12
		0.82	0.41	0.87	1.58	2.63	0.25	0.48	0.25	0.25	0.48	4.89	0.48	2.18	3.07Δ	4.98	0.24	0.71	0.63	0.48
	高剂量	1.50	3.00	5.00	5.88	26.00	4.12	0.88	0.62	1.00	0.25	48.25	1.00	7.88	28.62	37.50	1.29	9.50	2.38	1.12
		0.41	1.22	0.41	1.80	2.80	1.32	0.63	0.48	0.41	0.29	1.85	0.41	1.89	1.44Δ	1.78	0.10	1.47	0.85	0.48
停药恢复 4 周	对照组	1. 25	2. 50	5. 00	6. 50	27. 00	5. 00	0. 75	1. 00	0.50	0. 25	49. 75	1. 25	7. 25	30. 00	38. 50	1. 33	8. 00	2. 50	0. 75
		0. 35	0. 00	0. 00	0. 71	3. 54	2. 12	1. 06	0. 71	0.71	0. 35	8. 84	0. 35	0. 35	6. 36	6. 36	0. 45	1. 41	0. 00	1. 06
	低剂量	1. 25	2. 00	6. 25	7. 75	24. 25	5. 50	0. 75	1. 50	2. 50	0. 75	52. 50	1. 25	6. 25	28. 25	35. 75	1. 50	7. 75	1. 25	1. 25
		0. 35	0. 00	0. 35	0. 35	8. 84	0. 71	0. 35	0. 71	0. 71	0. 35	8. 48	0. 35	0. 35	5. 30	5. 30	0. 46	1. 77	1. 77	1. 06
	中剂量	0.75	1.75	4.75	8.00	27.00	4.75	0.75	1.25	1.50	0.50	51.00	1.75	5.75	27.00	34.50	1.49	9.75	2.00	1.25
		0.35	0.35	0.35	0.00	4.24	0.35	0.35	1.06	0.71	0.71	6.36	0.35	1.06	4.24	2.83	0.31	0.35	0.71	1.06
	高剂量	1.50	2.50	6.00	7.00	26.00	5.75	0.75	1.00	1.25	0.50	52.25	1.25	9.00	25.75	36.00	1.45	7.25	2.50	1.00
		0.71	0.00	0.71	0.00	0.71	1.06	0.35	0.00	0.35	0.00	1.77	0.35	1.41	2.48	0.71	0.08	0.35	0.71	0.00

方差分析：各组间比较无显著性差异（p>0.05）；

注：恢复期观察每组动物数仅为两只，故不再做统计学比较，仅以 $\bar{x}$ ±SD 表示；

Notes:

Variance analysis, there was no significant difference between the groups ($p > 0.05$).

During the recovery period, only two animals were observed in each group, so statistical comparison was no longer performed and was expressed as mean ± SD.

Table S27 Hematology results before and after TA administration via group comparison

表 28　Beagle 犬给药前和给药 5、9、13 周血液学各指标组间比较结果（$\bar{x}$ ±SD）

测定时间	组别	WBC	NE	LY	MO	RBC	Hb	RDW	MCV	MCH	MCHC	PLT	PT	RET
		($\times10^9$/L)	%	%	%	($\times10^{12}$/L)	(g/L)	(%)	(fL)	(pg)	(g/L)	($\times10^9$/L)	(Sec)	(%)
给药前 (n=6)	对照组	11.67±1.98	68.26±2.55	25.72±2.42	5.67±0.70	6.29±1.09	130.58±16.42	17.40±0.81	50.49±1.28	20.19±0.58	396.17±12.80	340.83±70.53	7.50±0.11	0.68±0.14
	低剂量	12.16±2.70	69.21±5.18	25.17±5.36	5.56±0.92	6.24±0.71	127.17±11.89	18.20±0.57	51.01±1.16	20.45±0.58	400.67±7.17	400.17±78.19	7.52±0.68	0.63±0.17
	中剂量	12.34±2.55	70.96±2.16	23.02±2.24	5.79±0.66	6.22±0.59	122.00±9.08	18.40±0.15	49.52±2.02	19.68±0.77	397.58±2.42	427.83±63.34	7.23±0.39	0.61±0.17
	高剂量	12.52±3.17	67.73±3.72	26.99±4.15	5.06±0.75	6.24±0.59	125.67±12.19	18.17±0.61	50.22±1.78	20.11±0.76	399.00±4.10	341.67±59.11	7.25±0.21	0.67±0.13
给药 5 周 (n=6)	对照组	13.82±3.34	66.55±3.40	27.31±3.57	5.91±0.87	7.13±0.82	149.67±21.44	17.08±0.95	49.88±1.36	20.75±1.10	399.00±33.49	362.67±65.62	5.97±0.22	0.65±0.28
	低剂量	12.26±3.65	70.01±4.97	24.92±4.90	5.01±0.48	6.75±1.07	140.33±19.37	17.03±0.77	50.62±1.38	20.82±0.66	412.17±11.89	359.33±62.64	5.92±0.43	0.57±0.26
	中剂量	12.04±2.27	70.55±2.81	24.52±2.68	4.85±0.77	6.49±0.35	131.00±8.17	17.35±0.77	48.47±1.94	20.20±0.80	415.17±8.66	394.00±34.20	5.88±0.28	0.60±0.21
	高剂量	13.88±3.62	67.52±4.39	26.98±4.95	5.43±0.92	6.68±0.75	137.00±16.82	16.98±0.66	50.28±1.63	20.48±0.66	409.00±4.94	310.67±47.79	5.98±0.31	0.60±0.22
给药 9 周 (n=6)	对照组	13.21±2.72	67.39±5.22	24.88±3.29	6.72±2.49	7.35±0.58	152.33±11.55	17.68±2.04	53.68±5.25	20.70±0.62	394.67±33.28	268.67±42.61	6.28±0.40	0.73±0.32
	低剂量	15.62±3.66	68.41±4.82	25.35±5.11	6.11±1.85	6.72±1.01	143.33±19.82	17.12±0.67	51.00±0.90	21.30±0.60	419.17±8.30	330.83±58.04	6.55±1.43	0.68±0.20
	中剂量	11.77±4.31	71.10±3.18	22.08±2.86	6.22±1.85	6.56±0.68	136.50±15.69	17.40±1.27	51.80±6.26	20.82±0.46	404.00±37.28	311.33±72.66	5.73±0.24	0.70±0.33
	高剂量	13.38±2.66	68.61±3.52	24.90±4.12	5.96±1.43	6.69±0.59	141.33±10.63	16.43±1.52	52.97±8.22	21.22±0.88	402.83±45.24	290.83±56.69	6.25±0.50	0.72±0.23
给药 13 周 (n=6)	对照组	13.00±3.49	68.65±2.54	25.47±2.77	5.74±0.94	7.30±0.75	154.00±12.46	16.67±0.38	50.30±0.73	21.12±0.81	419.00±11.54	306.00±40.83	6.42±0.38	0.85±0.44
	低剂量	16.09±5.89	69.48±6.85	24.28±7.12	5.81±0.47	7.03±0.62	147.67±10.98	16.80±0.39	51.03±1.27	21.08±0. 64	415.00±6.13	317.00±59.34	6.53±0.96	0.97±0.44
	中剂量	12.13±2.61	71.55±3.48	23.56±2.84	4.56±1.25	6.17±1.22	139.00±4.69*	17.18±0.82	49.82±1.41	20.88±0.56	418.33±6.02	366.00±19.22	6.38±0.90	0.60±0.18
	高剂量	13.46±2.87	66.92±3.43	27.17±3.77	5.44±0.81	6.62±0.18	140.33±2.66*	16.22±0.41▲▲	51.03±1.03	21.28±0.62	415.33±6.68	357.00±56.21	6.35±0.84	1.03±0.64

方差分析：*与空白对照组比较有显著性差异（p <0.05）；▲▲与中剂量组比较有显著性差异(p<0.01)；

注：　给药前各指标 $\bar{x}$ ±SD 为给药前第 1、2 周检测值平均值统计结果；

Notes:

Variance analysis, *p < 0.05 compared with the control group; ▲▲ p < 0.01 compared with the 60 mg/kg.bw group.

The data before administration represented the mean value at week -1 and -2 and was expressed as mean ± SD.

Table S28 Hematology results after TA withdrawal for 4 weeks via group comparison

表 29　Beagle 犬停药恢复 4 周血液学各指标组间比较结果（$\bar{x}$ ±SD）

测定时间	组别	WBC ($\times10^9$/L)	NE %	LY %	MO %	RBC ($\times10^{12}$/L)	Hb (g/L)	RDW (%)	MCV (fL)	MCH (pg)	MCHC (g/L)	PLT ($\times10^9$/L)	PT (Sec)	RET (%)
停药恢复 4 周 (n=2)	对照组	13.50±1.58	63.11±3.58	27.48±2.53	6.48±0.05	7.29±0.30	157.00±7.07	17.05±1.91	52.10±1.41	21.50±0.00	415.00±7.07	302.00±56.57	6.50±0.28	0.90±0.14
	低剂量	12.94±1.28	62.80±10.20	28.08±6.85	6.46±2.69	6.11±0.41	136.00±2.83	17.05±1.91	52.20±0.56	22.20±0.85	419.50±10.61	302.00±56.57	5.25±0.07	0.55±0.07
	中剂量	15.06±8.04	63.76±6.30	28.02±4.15	5.32±0.91	6.20±0.68	132.00±8.48	15.80±0.00	51.55±2.19	21.25±0.92	415.00±1.41	375.00±244.66	5.95±0.50	0.85±0.35
	高剂量	13.62±1.70	65.26±8.88	26.25±5.50	5.04±1.89	5.68±0.34	124.00±8.48	15.80±0.14	52.75±0.64	21.95±0.21	421.50±2.12	403.00±117.38	5.20±0.56	0.45±0.07

方差分析：各组间比较无显著性差异（p>0.05）；

注：1. 给药前各指标 $\bar{x}$ ±SD 为给药前第 1、2 周检测值平均值统计结果；

2. 恢复期观察每组动物数仅为两只，故不再做统计学比较，仅以 $\bar{x}$ ±SD 表示；

Notes:

Variance analysis, there was no significant difference between the groups ($p > 0.05$).

The data before administration represented the mean value at week -1 and -2 and was expressed as mean ± SD.

During the recovery period, only two animals were observed in each group, so statistical comparison was no longer performed and was expressed as mean ± SD.

Table S29 Hematology results before and after TA administration via self-comparison

表 30　Beagle 犬给药 5、9、13 周血液学各指标与给药前自身对照比较结果（$\bar{x}$ ±SD）

测定时间	组别	WBC (×10⁹/L)	NE %	LY %	MO %	RBC (×10¹²/L)	Hb (g/L)	RDW (%)
给药前 (n=6)	对照组	11.67±1.98	68.26±2.55	25.72±2.42	5.67±0.70	6.29±1.09	130.58±16.42	17.40±0.81
	低剂量	12.16±2.70	69.21±5.18	25.17±5.36	5.56±0.92	6.24±0.71	127.17±11.89	18.20±0.57
	中剂量	12.34±2.55	70.96±2.16	23.02±2.24	5.79±0.66	6.22±0.59	122.00±9.08	18.40±0.15
	高剂量	12.52±3.17	67.73±3.72	26.99±4.15	5.06±0.75	6.24±0.59	125.67±12.19	18.17±0.61
给药 5 周 (n=6)	对照组	13.82±3.34	66.55±3.40♥♥	27.31±3.57♥♥	5.91±0.87♥♥	7.13±0.82♥♥	149.67±21.44	17.08±0.95♥♥
	低剂量	12.26±3.65♥♥	70.01±4.97♥♥	24.92±4.90♥♥	5.01±0.48♥♥	6.75±1.07♥♥	140.33±19.37♥♥	17.03±0.77♥♥
	中剂量	12.04±2.27♥♥	70.55±2.81♥♥	24.52±2.68♥♥	4.85±0.77♥♥	6.49±0.35♥♥	131.00±8.17	17.35±0.77♥♥
	高剂量	13.88±3.62♥	67.52±4.39♥♥	26.98±4.95♥♥	5.43±0.92♥♥	6.68±0.75♥♥	137.00±16.82♥♥	16.98±0.66♥♥
给药 9 周 (n=6)	对照组	13.21±2.72	67.39±5.22♥♥	24.88±3.29♥♥	6.72±2.49	7.35±0.58♥	152.33±11.55	17.68±2.04♥♥
	低剂量	15.62±3.66♥	68.41±4.82♥♥	25.35±5.11♥♥	6.11±1.85♥	6.72±1.01♥♥	143.33±19.82♥♥	17.12±0.67♥♥
	中剂量	11.77±4.31♥♥	71.10±3.18♥♥	22.08±2.86♥♥	6.22±1.85♥	6.56±0.68♥♥	136.50±15.69	17.40±1.27♥♥
	高剂量	13.38±2.66♥	68.61±3.52♥♥	24.90±4.12♥	5.96±1.43	6.69±0.59♥♥	141.33±10.63♥♥	16.43±1.52♥♥
给药 13 周 (n=6)	对照组	13.00±3.49♥	68.65±2.54♥♥	25.47±2.77♥♥	5.74±0.94♥♥	7.30±0.75♥	154.00±12.46	16.67±0.38♥♥
	低剂量	16.09±5.89	69.48±6.85♥♥	24.28±7.12♥♥	5.81±0.47♥♥	7.03±0.62♥♥	147.67±10.98♥♥	16.80±0.39♥♥
	中剂量	12.13±2.61♥♥	71.55±3.48♥♥	23.56±2.84♥♥	4.56±1.25♥♥	6.17±1.22♥♥	139.00±4.69	17.18±0.82♥♥
	高剂量	13.46±2.87	66.92±3.43♥♥	27.17±3.77♥	5.44±0.81♥♥	6.62±0.18♥♥	140.33±2.66♥♥	16.22±0.41♥♥

配对 t 检验：♥/♥♥与给药前自身对照比较有显著性差异（p <0.05/0.01）；

注：　给药前各指标 $\bar{x}$ ±SD 为给药前第 1、2 周检测值平均值统计结果；

续表 30：

测定时间	组别	MCV (fL)	MCH (pg)	MCHC (g/L)	PLT (×10⁹/L)	PT (Sec)	RET (%)
给药前 (n=6)	对照组	50.49±1.28	20.19±0.58	396.17±12.80	340.83±70.53	7.50±0.11	0.68±0.14
	低剂量	51.01±1.16	20.45±0.58	400.67±7.17	400.17±78.19	7.52±0.68	0.63±0.17
	中剂量	49.52±2.02	19.68±0.77	397.58±2.42	427.83±63.34	7.23±0.39	0.61±0.17
	高剂量	50.22±1.78	20.11±0.76	399.00±4.10	341.67±59.11	7.25±0.21	0.67±0.13
给药 5 周 (n=6)	对照组	49.88±1.36♥♥	20.75±1.10♥♥	399.00±33.49♥♥	362.67±65.62♥	5.97±0.22♥♥	0.65±0.28
	低剂量	50.62±1.38♥♥	20.82±0.66♥♥	412.17±11.89♥♥	359.33±62.64♥♥	5.92±0.43♥♥	0.57±0.26♥♥
	中剂量	48.47±1.94♥♥	20.20±0.80♥♥	415.17±8.66♥♥	394.00±34.20♥♥	5.88±0.28♥♥	0.60±0.21
	高剂量	50.28±1.63♥♥	20.48±0.66♥♥	409.00±4.94♥♥	310.67±47.79♥♥	5.98±0.31♥♥	0.60±0.22
给药 9 周 (n=6)	对照组	53.68±5.25♥♥	20.70±0.62♥♥	394.67±33.28♥♥	268.67±42.61♥♥	6.28±0.40♥♥	0.73±0.3
	低剂量	51.00±0.90♥♥	21.30±0.60♥♥	419.17±8.30♥♥	330.83±58.04♥♥	6.55±1.43♥♥	0.68±0.20♥
	中剂量	51.80±6.26♥♥	20.82±0.46♥♥	404.00±37.28♥♥	311.33±72.66♥♥	5.73±0.24♥♥	0.70±0.33
	高剂量	52.97±8.22♥♥	21.22±0.88♥♥	402.83±45.24♥♥	290.83±56.69♥♥	6.25±0.50♥♥	0.72±0.23
给药 13 周 (n=6)	对照组	50.30±0.73♥♥	21.12±0.81♥♥	419.00±11.54♥♥	306.00±40.83♥♥	6.42±0.38♥♥	0.85±0.44
	低剂量	51.03±1.27♥♥	21.08±0. 64♥♥	415.00±6.13♥♥	317.00±59.34♥♥	6.53±0.96♥♥	0.97±0.44
	中剂量	49.82±1.41♥♥	20.88±0.56♥♥	418.33±6.02♥♥	366.00±19.22♥♥	6.38±0.90♥♥	0.60±0.18♥
	高剂量	51.03±1.03♥♥	21.28±0.62♥♥	415.33±6.68♥♥	357.00±56.21♥♥	6.35±0.84♥♥	1.03±0.64

配对 t 检验：♥/♥♥与给药前自身对照比较有显著性差异（p <0.05/0.01）；

注：　给药前各指标 $\bar{x}$ ±SD 为给药前第 1、2 周检测值平均值统计结果；

Notes:

Pairwise T-test: ♥/♥♥ $p < 0.05/0.01$ self-comparison with before administration.

The data before administration represented the mean value at week -1 and -2 and was expressed as mean ± SD.

Table S30 Hematology results after TA withdrawal via self-comparison

表 31 Beagle 犬停药恢复 4 周血液学各指标与给药前自身对照比较结果（$\bar{x}$ ±SD）

测定时间	组别	WBC	NE	LY	MO	RBC	Hb	RDW
		($\times10^9$/L)	%	%	%	($\times10^{12}$/L)	(g/L)	(%)
停药恢复 4 周 (n=2)	对照组	13.50±1.58	63.11±3.58♥	27.48±2.53♥	6.48±0.05	7.29±0.30	157.00±7.07	17.05±1.91
	低剂量	12.94±1.28	62.80±10.20	28.08±6.85	6.46±2.69	6.11±0.41	136.00±2.83	17.05±1.91
	中剂量	15.06±8.04	63.76±6.30♥	28.02±4.15♥♥	5.32±0.91	6.20±0.68	132.00±8.48	15.80±0.00♥
	高剂量	13.62±1.70	65.26±8.88	26.25±5.50	5.04±1.89♥	5.68±0.34	124.00±8.48	15.80±0.14

	组别	MCV	MCH	MCHC	PLT	PT	RET
		(fL)	(pg)	(g/L)	($\times10^9$/L)	(Sec)	(%)
停药恢复 4 周 (n=2)	对照组	52.10±1.41♥	21.50±0.00♥	415.00±7.07♥	302.00±56.57♥	6.50±0.28♥	0.90±0.14
	低剂量	52.20±0.56♥	22.20±0.85♥	419.50±10.61	302.00±56.57	5.25±0.07	0.55±0.07
	中剂量	51.55±2.19♥♥	21.25±0.92♥♥	415.00±1.41♥♥	375.00±244.66	5.95±0.50	0.85±0.35
	高剂量	52.75±0.64	21.95±0.21♥	421.50±2.12♥♥	403.00±117.38	5.20±0.56♥	0.45±0.07

配对 t 检验：♥/♥♥与给药前自身对照比较有显著性差异（p <0.05/0.01）；

注：1. 给药前各指标 $\bar{x}$ ±SD 为给药前第 1、2 周检测值平均值统计结果；

2. 恢复期观察每组动物数仅为两只，故不再做统计学比较，仅以 $\bar{x}$ ±SD 表示；

Notes:

Pairwise T-test: ♥/♥♥ $p < 0.05/0.01$ self-comparison with before administration.

The data before administration represented the mean value at week -1 and -2 and was expressed as mean ± SD.

During the recovery period, only two animals were observed in each group, so statistical comparison was no longer performed and was expressed as mean ± SD.

Table S31 Biochemistry results after TA administration via group comparison

表 37 Beagle 犬给药前和给药 5、9、13 周血清生化学各指标组间比较结果（$\bar{x}$ ±SD）

测定时间	组别	ALT (U/L)	AST (U/L)	TG (mmol/L)	TC (mmol/L)	GLU (mmol/L)	Cre (μmol/L)	Bun (mmol/L)	GGT (U/L)	CK (U/L)	ALB (g/L)	TP (g/L)	ALP (U/L)	TBIL (μmol/L)
给药前 (n=6)	对照组	25.00±2.76	35.50±4.07	0.40±0.24	4.29±0.46	4.62±0.14	113.33±9.11	3.74±0.72	4.17±1.08	268.25±46.57	30.12±1.36	73.98±8.12	146.00±16.90	6.00±0.83
	低剂量	22.75±3.63	35.33±2.38	0.34±0.06	4.45±0.58	4.64±0.46	114.92±12.64	3.94±0.93	3.83±1.66	248.83±59.78	29.30±1.82	81.47±8.09	139.33±7.30	5.14±1.38
	中剂量	22.83±3.37	33.83±4.19	0.34±0.07	4.55±0.45	4.83±0.29	106.58±12.38	3.66±0.36	3.67±2.16	239.92±48.49	29.60±0.82	80.03±14.40	137.17±8.72	6.61±2.34
	高剂量	24.25±5.49	36.83±4.59	0.36±0.07	5.19±1.32	4.51±0.20	94.58±22.35	3.55±0.76	3.50±1.26	281.25±44.76	29.69±1.94	99.06±36.54	142.83±12.44	5.08±1.22
给药 5 周 (n=6)	对照组	27.17±5.04	37.83±2.56	0.43±0.26	4.04±0.30	4.85±0.33	108.33±15.62	3.98±0.26	3.83±1.60	229.33±41.18	31.17±1.52	80.72±4.32	158.17±53.27	5.18±0.92
	低剂量	25.17±7.55	34.17±5.04	0.50±0.16	4.15±0.49	4.50±0.21	100.33±21.60	4.22±1.06	3.67±1.63	189.33±29.79	31.30±3.37	85.10±4.28	141.00±33.17	4.87±1.17
	中剂量	22.00±4.34	35.83±8.18	0.47±0.08	4.30±0.35	4.67±0.46	102.50±7.26Δ	4.07±0.26	2.83±1.83	229.50±97.19	29.98±1.38	85.98±24.94	152.33±52.71	4.95±0.86
	高剂量	28.00±8.62	39.83±7.44	0.75±0.29	4.46±0.49	4.42±0.42	110.17±24.10Δ	4.28±0.90	3.50±2.17	239.67±69.44	29.53±2.16	85.08±15.83	160.50±30.45	6.07±1.54
给药 9 周 (n=6)	对照组	31.50±8.19	39.00±2.28	0.48±0.23	4.35±0.33	4.92±0.36	114.67±7.17	4.38±0.45	2.17±0.75	208.67±38.51	30.95±0.75	85.80±2.83	138.00±34.80	6.12±1.24
	低剂量	26.17±3.82	35.17±2.79	0.58±0.18	4.98±1.53	4.72±0.36	118.67±24.54	4.07±0.53	3.00±1.26	182.17±61.95	30.40±2.00	86.90±9.07	146.17±36.04	6.40±2.05
	中剂量	32.33±9.52	39.17±9.89	0.50±0.17	4.80±2.03	4.67±0.67	113.67±28.88	4.28±0.43	3.00±1.10	177.67±36.70	29.75±2.48	81.77±5.26	140.50±23.11	6.20±1.53
	高剂量	38.83±17.38	42.33±4.76	0.76±0.40	5.08±1.38	4.62±0.50	101.67±11.16	4.18±0.32	2.50±1.05	193.67±57.51	30.15±1.07	90.25±11.40	145.17±24.60	5.90±1.77
给药 13 周 (n=6)	对照组	31.17±4.96	39.67±2.34	0.54±0.24	0.27±0.04	4.22±0.42	148.33±14.46	4.65±0.29	2.50±1.52	224.67±35.08	31.60±0.81	89.98±5.77	130.17±44.22	6.38±1.40
	低剂量	27.00±7.24	36.50±3.51	0.54±0.16	0.29±0.05	4.02±0.20	145.33±22.33	4.78±0.56	3.00±0.89	199.67±31.02	30.30±1.68	89.72±3.52	131.33±22.18	6.55±1.61
	中剂量	29.17±8.13	37.00±4.20	0.45±0.07	0.29±0.04	4.05±0.36	151.00±19.95	4.48±0.46	2.83±1.47	192.33±38.64	29.42±1.38**	81.88±6.64	116.17±18.15	6.90±1.47
	高剂量	33.67±6.62	44.17±7.81	0.64±0.28	0.35±0.12	3.80±0.35	128.83±12.73	4.57±0.22	3.83±1.33	205.17±33.02	28.90±1.05**	81.77±9.92	137.33±45.30	5.82±1.04

方差分析：**与空白对照组比较有显著性差异（p <0.01）；

Notes:

Variance analysis, *p < 0.01 compared with the control group;

Table S32 Biochemistry results after TA withdrawal for 4 weeks via group comparison

表 38　Beagle 犬停药恢复 4 周血清生化学各指标组间比较结果（$\bar{x}$ ±SD）

测定时间	组别	ALT (U/L)	AST (U/L)	TG (mmol/L)	TC (mmol/L)	GLU (mmol/L)	Cre (μmol/L)	Bun (mmol/L)	GGT (U/L)	CK (U/L)	ALB (g/L)	TP (g/L)	ALP (U/L)	TBIL (μmol/L)
停药恢复 4 周 (n=2)	对照组	29.50±6.36	40.50±12.02	0.47±0.24	4.48.±0.14	4.45±0.21	151.00±7.07	4.05±0.21	5.00±1.41	146.50±34.65	34.50±0.14	89.15±11.95	102.00±32.53	4.00±1.41
	低剂量	24.00±7.07	34.00±5.66	0.70±0.32	4.10±0.52	4.60±0.28	169.50±17.68	4.45±1.20	3.00±1.41	196.00±53.74	34.65±0.35	80.35±5.73	106.50±9.19	6.25±2.33
	中剂量	26.00±1.41	29.50±9.19	0.33±0.16	4.34±0.00	4.70±0.56	147.50±7.78	3.80±0.14	4.00±2.83	99.50±4.95	35.10±0.42	79.45±3.75	85.00±12.73	4.20±0.56
	高剂量	29.50±9.19	35.00±2.83	0.48±0.27	4.80±0.37	5.00±0.14	146.50±7.78	4.15±0.07	3.50±2.12	172.50±12.02	34.65±0.35	99.80±5.94	97.00±11.31	3.25±2.33

方差分析：各组间比较无显著性差异（p>0.05）；

注：1. 给药前各指标的 $\bar{x}$ ±SD 为给药前第 1、2 周检测值平均值统计结果；

2. 恢复期观察每组动物数仅为两只，故不再做统计学比较，仅以 $\bar{x}$ ±SD 表示；

Notes:

Variance analysis, there was no significant difference between the groups ($p > 0.05$).

The data before administration represented the mean value at week -1 and -2 and was expressed as mean ± SD.

During the recovery period, only two animals were observed in each group, so statistical comparison was no longer performed and was expressed as mean ± SD.

Table S33 Biochemistry results after TA administration via self-comparison

表 39　Beagle 犬给药 5、9、13 周血清生化学各指标与给药前自身对照比较结果（$\bar{x}$ ±SD）

测定时间	组别	ALT (U/L)	AST (U/L)	TG (mmol/L)	TC (mmol/L)	GLU (mmol/L)	Cre (μmol/L)	Bun (mmol/L)
给药前 (n=6)	对照组	25.00±2.76	35.50±4.07	0.40±0.24	4.29±0.46	4.62±0.14	113.33±9.11	3.74±0.72
	低剂量	22.75±3.63	35.33±2.38	0.34±0.06	4.45±0.58	4.64±0.46	114.92±12.64	3.94±0.93
	中剂量	22.83±3.37	33.83±4.19	0.34±0.07	4.55±0.45	4.83±0.29	106.58±12.38	3.66±0.36
	高剂量	24.25±5.49	36.83±4.59	0.36±0.07	5.19±1.32	4.51±0.20	94.58±22.35	3.55±0.76
给药 5 周 (n=6)	对照组	27.17±5.04	37.83±2.56	0.43±0.26	4.04±0.30	4.85±0.33	108.33±15.62	3.98±0.26
	低剂量	25.17±7.55	34.17±5.04	0.50±0.16♥	4.15±0.49	4.50±0.21	100.33±21.60	4.22±1.06
	中剂量	22.00±4.34	35.83±8.18	0.47±0.08♥♥	4.30±0.35	4.67±0.46	102.50±7.26	4.07±0.26
	高剂量	28.00±8.62	39.83±7.44	0.75±0.29♥	4.46±0.49	4.42±0.42	110.17±24.10	4.28±0.90
给药 9 周 (n=6)	对照组	31.50±8.19	39.00±2.28	0.48±0.23	4.35±0.33	4.92±0.36	114.67±7.17	4.38±0.45♥
	低剂量	26.17±3.82♥♥	35.17±2.79	0.58±0.18♥	4.98±1.53	4.72±0.36	118.67±24.54	4.07±0.53
	中剂量	32.33±9.52	39.17±9.89	0.50±0.17♥	4.80±2.03	4.67±0.67	113.67±28.88	4.28±0.43
	高剂量	38.83±17.38♥	42.33±4.76♥♥	0.76±0.40	5.08±1.38	4.62±0.50	101.67±11.16	4.18±0.32
给药 13 周 (n=6)	对照组	31.17±4.96	39.67±2.34♥	0.54±0.24♥	0.27±0.04♥♥	4.22±0.42	148.33±14.46♥♥	4.65±0.29♥♥
	低剂量	27.00±7.24	36.50±3.51	0.54±0.16♥	0.29±0.05♥♥	4.02±0.20♥♥	145.33±22.33♥	4.78±0.56♥
	中剂量	29.17±8.13	37.00±4.20♥	0.45±0.07♥	0.29±0.04♥♥	4.05±0.36♥	151.00±19.95♥♥	4.48±0.46♥
	高剂量	33.67±6.62♥	44.17±7.81	0.64±0.28♥	0.35±0.12♥♥	3.80±0.35♥♥	128.83±12.73♥♥	4.57±0.22♥

配对 t 检验：♥/♥♥与给药前自身对照比较有显著性差异（p <0.05/0.01）；

注：给药前各指标的 $\bar{x}$ ±SD 为给药前第 1、2 周检测值平均值统计结果；

续表 39：

测定时间	组别	GGT (U/L)	CK (U/L)	ALB (g/L)	TP (g/L)	ALP (U/L)	TBIL (μmol/L)
给药前 (n=6)	对照组	4.17±1.08	268.25±46.57	30.12±1.36	73.98±8.12	146.00±16.90	6.00±0.83
	低剂量	3.83±1.66	248.83±59.78	29.30±1.82	81.47±8.09	139.33±7.30	5.14±1.38
	中剂量	3.67±2.16	239.92±48.49	29.60±0.82	80.03±14.40	137.17±8.72	6.61±2.34
	高剂量	3.50±1.26	281.25±44.76	29.69±1.94	99.06±36.54	142.83±12.44	5.08±1.22
给药 5 周 (n=6)	对照组	3.83±1.60	229.33±41.18	31.17±1.52	80.72±4.32	158.17±53.27	5.18±0.92
	低剂量	3.67±1.63	189.33±29.79	31.30±3.37♥	85.10±4.28	141.00±33.17	4.87±1.17
	中剂量	2.83±1.83	229.50±97.19	29.98±1.38	85.98±24.94	152.33±52.71	4.95±0.86
	高剂量	3.50±2.17	239.67±69.44	29.53±2.16	85.08±15.83	160.50±30.45	6.07±1.54
给药 9 周 (n=6)	对照组	2.17±0.75♥♥	208.67±38.51♥♥	30.95±0.75	85.80±2.83♥	138.00±34.80	6.12±1.24
	低剂量	3.00±1.26	182.17±61.95	30.40±2.00	86.90±9.07	146.17±36.04	6.40±2.05
	中剂量	3.00±1.10	177.67±36.70♥	29.75±2.48	81.77±5.26	140.50±23.11	6.20±1.53
	高剂量	2.50±1.05	193.67±57.51♥♥	30.15±1.07	90.25±11.40	145.17±24.60	5.90±1.77
给药 13 周 (n=6)	对照组	2.50±1.52	224.67±35.08♥	31.60±0.81♥	89.98±5.77♥	130.17±44.22	6.38±1.40
	低剂量	3.00±0.89	199.67±31.02	30.30±1.68	89.72±3.52	131.33±22.18	6.55±1.61
	中剂量	2.83±1.47	192.33±38.64	29.42±1.38	81.88±6.64	116.17±18.15	6.90±1.47
	高剂量	3.83±1.33	205.17±33.02♥♥	28.90±1.05	81.77±9.92	137.33±45.30	5.82±1.04

配对 t 检验：♥/♥♥与给药前自身对照比较有显著性差异（p <0.05/0.01）；

注：给药前各指标的 $\bar{x}$ ±SD 为给药前第 1、2 周检测值平均值统计结果；

Notes:

Pairwise T-test: ♥/♥♥ $p < 0.05/0.01$ self-comparison with before administration.

The data before administration represented the mean value at week -1 and -2 and was expressed as mean ± SD.

Table S34 Biochemistry results after TA withdrawal for 4 weeks via self-comparison

表 40　Beagle 犬停药恢复 4 周血清生化学各指标与给药前自身对照比较结果（$\bar{x}$ ±SD）

测定时间	组别	ALT (U/L)	AST (U/L)	TG (mmol/L)	TC (mmol/L)	GLU (mmol/L)	Cre (μmol/L)	Bun (mmol/L)
停药恢复 4 周 (n=2)	对照组	29.50±6.36	40.50±12.02	0.47±0.24	4.48.±0.14	4.45±0.21	151.00±7.07♥	4.05±0.21
	低剂量	24.00±7.07	34.00±5.66	0.70±0.32	4.10±0.52	4.60±0.28	169.50±17.68♥	4.45±1.20
	中剂量	26.00±1.41	29.50±9.19	0.33±0.16	4.34±0.00	4.70±0.56	147.50±7.78	3.80±0.14
	高剂量	29.50±9.19	35.00±2.83	0.48±0.27	4.80±0.37	5.00±0.14	146.50±7.78	4.15±0.07

测定时间	组别	GGT (U/L)	CK (U/L)	ALB (g/L)	TP (g/L)	ALP (U/L)	TBIL (μmol/L)
停药恢复 4 周 (n=2)	对照组	5.00±1.41	146.50±34.65	34.50±0.14	89.15±11.95	102.00±32.53	4.00±1.41♥
	低剂量	3.00±1.41	196.00±53.74	34.65±0.35♥	80.35±5.73	106.50±9.19	6.25±2.33
	中剂量	4.00±2.83	99.50±4.95	35.10±0.42	79.45±3.75	85.00±12.73	4.20±0.56
	高剂量	3.50±2.12	172.50±12.02	34.65±0.35	99.80±5.94	97.00±11.31	3.25±2.33

配对 t 检验：♥/♥♥与给药前自身对照比较有显著性差异（p <0.05/0.01）；

注：1. 给药前各指标的 $\bar{x}$ ±SD 为给药前第 1、2 周检测值平均值统计结果；

2. 恢复期观察每组动物数仅为两只，故不再做统计学比较，仅以 $\bar{x}$ ±SD 表示；

Notes:

Pairwise T-test: ♥/♥♥ p < 0.05/0.01 self-comparison with before administration.

The data before administration represented the mean value at week -1 and -2 and was expressed as mean ± SD.

During the recovery period, only two animals were observed in each group, so statistical comparison was no longer performed and was expressed as mean ± SD.

Table S35 Electrolytes results during the study period via group- and self- comparison

表 46 Beagle 犬给药前后不同时期血清电解质组间和自身对照比较结果（$\bar{x}$ ±SD）

测定时间	组别	K^+ (mmol/L)	Na^+ (mmol/L)	CL^- (mmol/L)	iCa (mmol/L)	TCa (mmol/L)	pH
给药前 (n=6)	对照组	4.57±0.26	143.30±0.99	105.20±1.65	0.86±0.04	1.67±0.08	7.56±0.01
	低剂量	4.70±0.20	142.13±1.36	105.14±1.42	0.84±0.03	1.64±0.06	7.56±0.03
	中剂量	4.67±0.11	143.15±1.06	105.57±1.12	0.84±0.03	1.64±0.05	7.56±0.01
	高剂量	4.54±0.35	142.92±1.04	105.39±0.85	0.83±0.01	1.62±0.02	7.54±0.02
给药 5 周 (n=6)	对照组	4.31±0.13	142.07±7.59	108.23±3.58	1.31±0.03♥♥	2.55±0.05♥♥	7.49±0.02♥♥
	低剂量	4.54±0.22	145.10±0.95♥♥	109.92±1.69♥♥	1.29±0.03♥♥	2.52±0.06♥♥	7.49±0.008♥♥
	中剂量	4.45±0.16♥	144.23±0.66♥	109.52±0.97♥♥	1.31±0.02♥♥	2.55±0.04♥♥	7.49±0.008♥♥
	高剂量	4.55±0.14	144.57±0.94♥♥	110.50±1.42♥♥	1.30±0.03♥♥	2.54±0.07♥♥	7.48±0.01♥♥
给药 9 周 (n=6)	对照组	4.26±0.12♥	139.62±1.25♥♥	106.23±1.83	1.37±0.04♥♥	2.67±0.09♥♥	7.50±0.01♥♥
	低剂量	4.31±0.17♥	138.73±1.97♥♥	106.05±1.78	1.36±0.05♥♥	2.66±0.08♥♥	7.51±0.02♥♥
	中剂量	4.18±0.19♥♥	138.82±1.58♥♥	105.15±0.95	1.36±0.03♥♥	2.65±0.06♥♥	7.50±0.008♥♥
	高剂量	4.44±0.32	138.15±0.86♥♥	105.95±1.47	1.37±0.02♥♥	2.66±0.04♥♥	7.51±0.02♥♥
给药 13 周 (n=6)	对照组	4.48±0.26	138.82±1.16♥♥	112.00±2.30♥♥	1.33±0.04♥♥	2.59±0.08♥♥	7.49±0.01♥♥
	低剂量	4.56±0.28	138.95±1.42♥♥	111.10±2.28♥♥	1.35±0.05♥♥	2.64±0.10♥♥	7.49±0.01♥♥
	中剂量	4.62±0.13	138.68±0.80♥♥	111.50±1.86♥♥	1.34±0.02♥♥	2.62±0.04♥♥	7.49±0.005♥♥
	高剂量	4.62±0.35	138.58±1.42♥♥	111.20±0.96♥♥	1.34±0.01♥♥	2.61±0.03♥	7.48±0.01♥♥
停药恢复 4 周 (n=2)	对照组	4.10±0.007	125.95±1.20♥	97.80±2.97♥	1.27±0.03♥♥	2.48±0.06♥♥	7.49±0.01
	低剂量	4.24±0.21	125.75±0.07	98.70±2.97	1.28±0.06	2.49±0.13	7.50±0.01♥
	中剂量	4.00±0.26	126.50±0.00♥	99.50±0.28♥	1.30±0.02	2.52±0.04♥	7.50±0.007
	高剂量	3.93±0.25	127.15±0.92♥	100.75±0.21♥	1.31±0.00♥	2.55±0.01♥♥	7.50±0.007

方差分析：各组间比较无显著性差异（p>0.05）；

配对 t 检验：♥/♥♥与给药前自身对照比较有显著性差异（p <0.05/0.01）；

注：1. 给药前各指标的 $\bar{x}$ ±SD 为给药前第 1、2 次检测值平均值的统计结果；

2. 恢复期观察每组动物数仅为两只，故不再做统计学比较，仅以 $\bar{x}$ ±SD 表示；

Notes:

Variance analysis, there was no significant difference between the groups ($p > 0.05$).

Pairwise T-test: ♥/♥♥ $p < 0.05/0.01$ self-comparison with before administration.

The data before administration represented the mean value at week -1 and -2 and was expressed as mean ± SD.

During the recovery period, only two animals were observed in each group, so statistical comparison was no longer performed and was expressed as mean ± SD.

Table S36 Organ coefficients during the study period via group comparison

表 53　Beagle 犬给药 13 周和停药恢复 4 周各组脏器系数组间比较结果（$\bar{x}$ ±SD，%）

检测时间	组别	脑	心	肝	脾	肺	左肾	右肾	肾上腺	胸腺	子宫	卵巢	左睾丸	右睾丸	附睾
给药13周	对照组	0.81±0.15	0.86±0.06	2.94±0.32	0.26±0.01	0.78±0.10	0.24±0.02	0.22±0.02	0.01±0.002	0.11±0.002	0.04±0.004	0.010±0.003	0.06±0.003	0.06±0.01	0.03±0.002
	低剂量	0.81±0.07	0.76±0.04	2.93±0.14	0.22±0.05	0.66±0.09	0.22±0.03	0.22±0.03	0.01±0.003	0.07±0.02	0.21±0.06 **▲▲▽▽	0.02±0.0003 **▲▲▽▽	0.07±0.01	0.08±0.01	0.03±0.0003
	中剂量	0.82±0.08	0.82±0.06	3.03±0.12	0.22±0.03	0.78±0.01	0.22±0.02	0.22±0.02	0.008±0.003	0.07±0.03	0.01±0.002	0.005±0.0006	0.06±0.004	0.06±0.006	0.03±0.0007
	高剂量	0.80±0.04	0.80±0.04	3.30±0.28	0.27±0.01	0.73±0.07	0.26±0.03	0.25±0.02	0.01±0.002	0.08±0.03	0.03±0.02	0.008±0.004	0.06±0.03	0.06±0.03	0.03±0.004
停药4周	对照组	0.84±0.03	0.86±0.13	2.74±0.31	0.26±0.01	0.62±0.06	0.22±0.03	0.22±0.03	0.009±0.002	0.05±0.02	0.02±0.03	0.005±0.007	0.03±0.05	0.13±0.18	0.29±0.41
	低剂量	0.72±0.13	0.80±0.03	2.74±0.13	0.25±0.02	0.67±0.07	0.22±0.03	0.21±0.03	0.009±0.001	0.12±0.04	0.03±0.05	0.005±0.007	0.04±0.05	0.04±0.05	0.01±0.02
	中剂量	0.76±0.03	0.83±0.05	2.66±0.33	0.23±0.02	0.68±0.04	0.22±0.01	0.22±0.007	0.01±0.003	0.04±0.01	0.01±0.02	0.003±0.004	0.04±0.05	0.03±0.05	0.02±0.02
	高剂量	0.80±0.13	0.87±0.02	3.32±0.13	0.26±0.04	0.78±0.08	0.29±0.05	0.26±0.006	0.01±0.002	0.04±0.02	0.07±0.10	0.008±0.01	0.03±0.05	0.03±0.04	0.02±0.02

方差分析：**与空白对照组比较有显著性差异（p<0.01）；▲▲与中剂量组比较有显著性差异(p<0.01)；▽▽与高剂量组比较有显著性差(p<0.01)；

注：恢复期观察每组动物数仅两只，故不再统计比较，仅以 $\bar{x}$ ±SD 表示。

Notes:

Variance analysis, **$p < 0.01$ compared with the control group; ▲▲ $p < 0.01$ compared with the 60 mg/kg.bw group; ▽▽ $p < 0.01$ compared with the 120 mg/kg.bw group.

During the recovery period, only two animals were observed in each group, so statistical comparison was no longer performed and was expressed as mean ± SD.

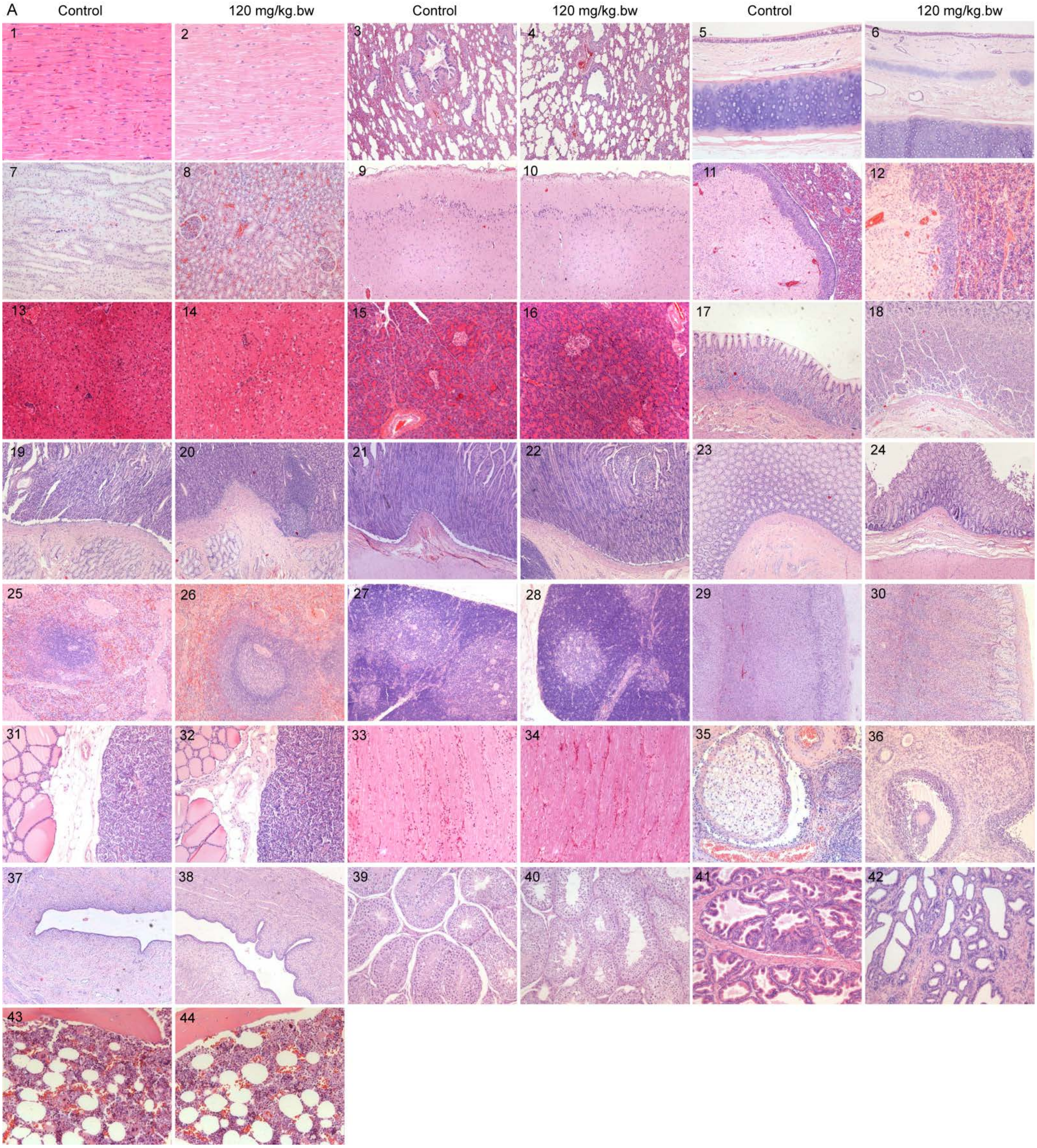

Fig. S2A. Representative sections of dogs in respect of 13 weeks treatment demonstrating histopathology

1 and **2** denoted the normal heart architectures of dogs in control and 120 mg/kg.bw group (× 20 magnification).

3 and **4** showed represented control and 120 mg/kg.bw dog lungs showing local interstitial pneumonia (× 5 magnification).

5 and **6** expressed the normal trachea of dogs in control and 120 mg/kg.bw group (× 10 magnification).

7 and **8** meant the control kidney accompanied by calcium deposit and 120 mg/kg.bw group was noemal (× 10 magnification).

9 and **10** represented the normal cerebral cortex of dogs in control and 120 mg/kg.bw group (× 10 magnification).

11 and **12** expressed the normal pituitary glands of dogs in control and 120 mg/kg.bw group (× 10 magnification).

13 and **14** denoted the control and 120 mg/kg.bw dog livers illuminating hepatocytes punctate necrotic (× 10 magnification).

15 and **16** represented the normal pancreases of dogs in control and 120 mg/kg.bw group (× 10 magnification).

17 and **18** represented the normal stomachs of dogs in control and 120 mg/kg.bw group (× 5 magnification).

19 and **20** the normal duodenums of dogs in control and 120 mg/kg.bw group (× 5 magnification).

21 and **22** denoted expressed the normal small intestine of dogs in control and 120 mg/kg.bw group (× 5 magnification).

23 and **24** represented the normal colons of dogs in control and 120 mg/kg.bw group (× 5 magnification).

25 and **26** showed the normal spleens of dogs in control and 120 mg/kg.bw group (× 10 magnification).

27 and **28** demonstrated the normal thymus glands of dogs in control and 120 mg/kg.bw group (× 10 magnification).

29 and **30** expressed the normal adrenal thymus glands of dogs in control and 120 mg/kg.bw group (× 5 magnification).

31 and **32** meant the normal thyroid and parathyroid glands of dogs in control and 120 mg/kg.bw group (× 5 magnification).

33 and **34** represented the normal optic nerves of dogs in control and 120 mg/kg.bw group (× 10 magnification).

35 and **36** showed the normal ovaries of dogs in control and 120 mg/kg.bw group (× 10 magnification).

37 and **38** expressed the normal uteruses of dogs in control and 120 mg/kg.bw group (× 5 magnification).

39 and **40** represented the normal testis of dogs in control and 120 mg/kg.bw group (× 5 magnification).

41 and **42** denoted the normal prostate glands of dogs in control and 120 mg/kg.bw rat (× 10 magnification).

43 and **44** meant the normal bone marrows of dogs in control and 120 mg/kg.bw group (× 20 magnification).

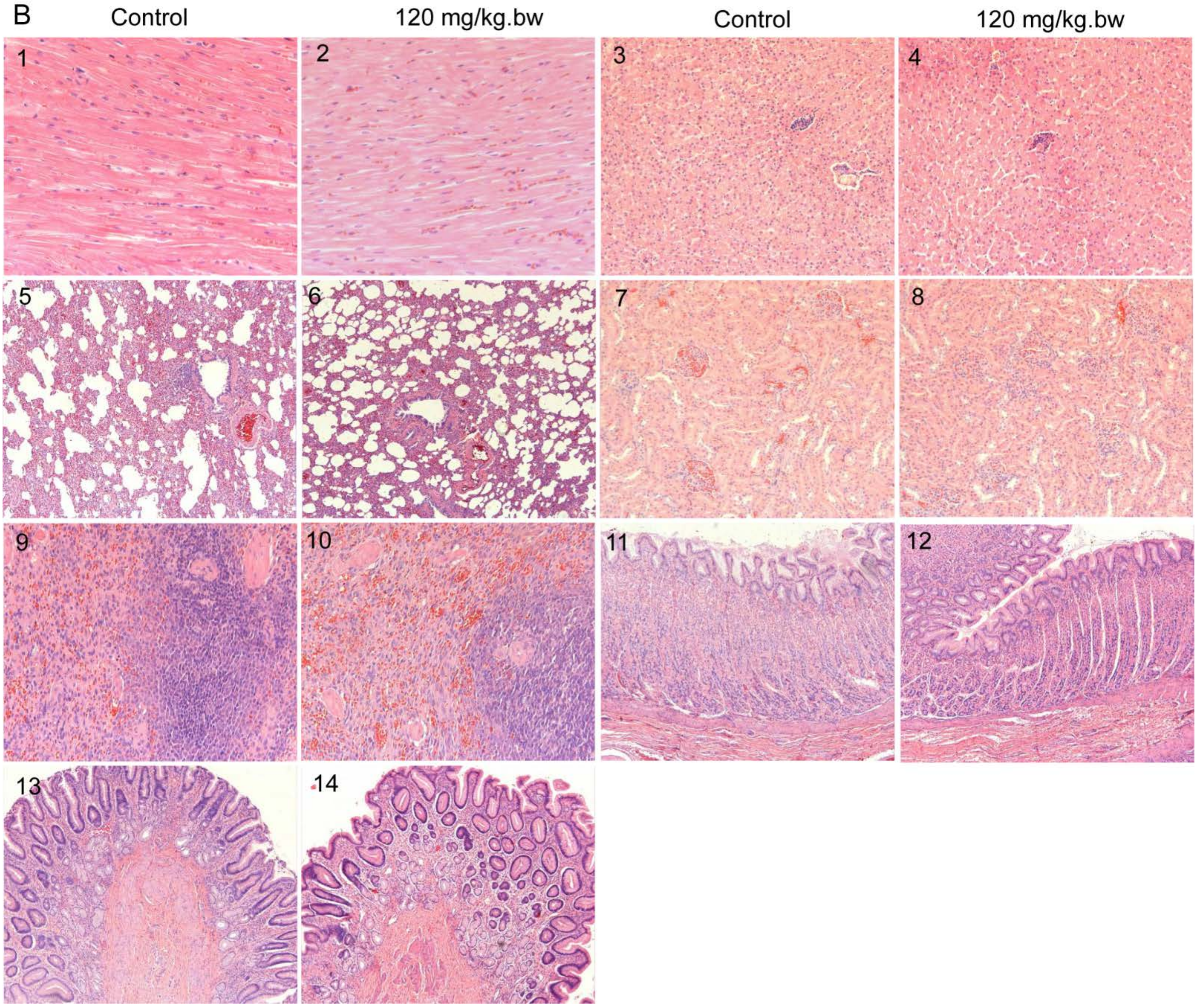

Fig. S2B. Representative sections of dogs in the recovery period demonstrating histopathology

1 and **2** denoted the normal heart architectures of dogs in control and 120 mg/kg.bw group (× 20 magnification).

3 and **4** represented the control and 120 mg/kg.bw dog livers illuminating hepatocytes small focal inflammation (× 10 magnification).

5 and **6** showed represented control and 120 mg/kg.bw dog lungs showing local interstitial pneumonia (× 5 magnification).

7 and **8** denoted the normal kidneys of dogs in control and 120 mg/kg.bw group (× 10 magnification).

9 and **10** denoted the normal spleens of dogs in control and 120 mg/kg.bw group (× 20 magnification).

11 and **12** denoted the normal stomachs of dogs in control and 120 mg/kg.bw group (× 20 magnification).

13 and **14** denoted the normal duodenums of dogs in control and 120 mg/kg.bw group (× 5 magnification).

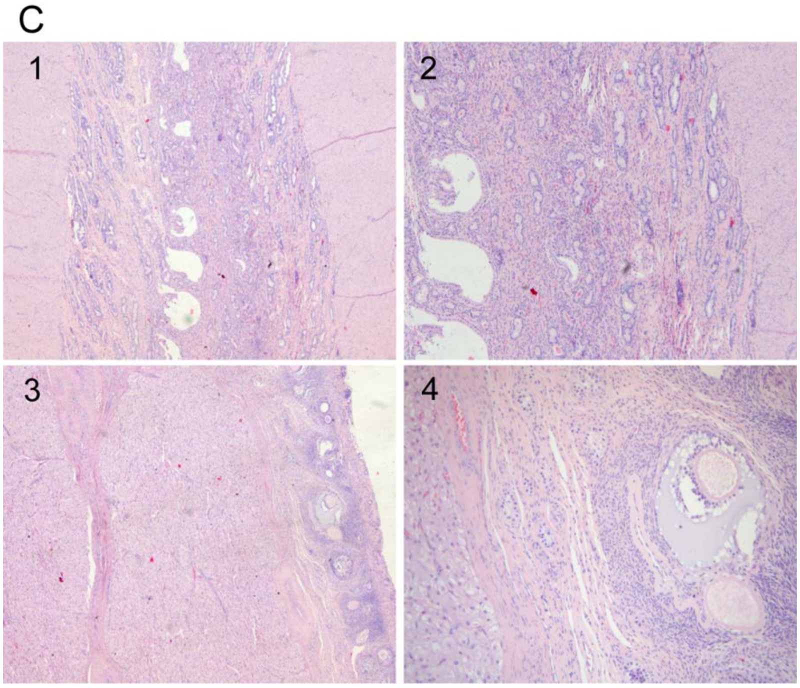

Fig. S2C. Representative uterus and ovary sections of two female dogs in the 20 mg/kg.bw at week 13

1 and **2** showed thickened endometrium, increased glands, and secretions in the gland cavity.

3 and **4** represented multiple large mature luteums.

Reference

1. X.H. Cai, Z.Z. Du, X.D. Luo. Org. Lett. **9**, 1817-1820 (2007)
2. X.W. Yang, X.J. Qin, Y.L. Zhao, P.K. Lunga, X.N. Li, S.Z. Jiang, G.G. Cheng, Y.P. Liu, X.D. Luo. Tetrahedron Lett. **55**, 4593-4596 (2014)
3. X.W. Yang, C.P. Yang, L.P. Jiang, X.J. Qin, Y.P. Liu, Q.S. Shen, Y.B. Chen, X.D. Luo. Org. Lett. **16**, 5808-5811 (2014)
4. X.H. Cai, Q.G. Tan, Y.P. Liu, T. Feng, Z.Z. Du, W.Q. Li, X.D. Luo. Org. Lett. **10**, 577-580 (2008)
5. X.W. Yang, C.W. Song, Y. Zhang, A. Khan, L.P. Jiang, Y.B. Chen, Y.P. Liu, X.D. Luo. Tetrahedron Lett. **56**, 6715-6718 (2015)
6. X.H. Cai, M.F. Bao, Y. Zhang, C.X. Zeng, Y.P. Liu, X.D. Luo. Org. Lett. **13**, 3568-3571 (2011)
7. Z.Q. Pan, X.J. Qin, Y.P. Liu, T. Wu, X.D. Luo, C.F. Xia. Org. Lett. **18**, 654-657 (2016)
8. M.W. Smith, S.A. Snyder. J. Am. Chem. Soc. **135**, 12964-12967 (2013)
9. G.L. Adams, P.J. Carroll, A.B. Smith. J. Am. Chem. Soc. **135**, 519-528 (2013)
10. G.L. Adams, P.J. Carroll, A.B. Smith. J. Am. Chem. Soc. **134**, 4037-4040 (2012)
11. T. Watanabe, N. Kato, N. Umezawa, T. Higuchi. Chem. Eur. J. **19**, 4255-4261 (2013)
12. D. Wang, M. Hou, Y. Ji, S.H. Gao. Org. Lett. **19**, 1922-1925 (2017)
13. T. Gerfaud, C.S. Xie, L. Neuville, J.P. Zhu. Angew. Chem., Int. Ed. **50**, 3954-3957 (2011)
14. J.D. Mason, S.M. Weinreb. The Journal of Organic Chemistry **83**, 5877-5896 (2018)

15. J. Moreno, E. Picazo, L.A. Morrill, J.M. Smith, N.K. Garg. J. Am. Chem. Soc. **138**, 1162-1165 (2016)
16. W.W. Ren, Q. Wang, J.P. Zhu. Angew. Chem., Int. Ed. **55**, 3500-3503 (2016)
17. Z.R. Xu, Q. Wang, J.P. Zhu. J. Am. Chem. Soc. **137**, 6712-6724 (2015)
18. Y. Yang, Y. Bai, S. Sun, M. Dai. Org. Lett. **16**, 6216-9 (2014)
19. A. Umehara, H. Ueda, H. Tokuyama. Org. Lett. **16**, 2526-2529 (2014)
20. K. Higuchi, S. Suzuki, R. Ueda, N. Oshima, E. Kobayashi, M. Tayu, T. Kawasaki. Org. Lett. **17**, 154-157 (2015)
21. J.M. Smith, J. Moreno, B.W. Boal, N.K. Garg. J. Org. Chem. **80**, 8954-8967 (2015)
22. J.M. Smith, J. Moreno, B.W. Boal, N.K. Garg. J. Am. Chem. Soc. **136**, 4504-4507 (2014)
23. X. Liang, S.Z. Jiang, K. Wei, Y.R. Yang. J. Am. Chem. Soc. **138**, 2560-2562 (2016)
